# Supplementary material for: An Insulin-Sensitive Circular RNA that Regulates Lifespan in Drosophila
Source: Mol Cell. 2020 Jul 16;79(2):268–279.e5. doi: 10.1016/j.molcel.2020.06.011 (PMC7318944; doi:10.1016/j.molcel.2020.06.011)
Supplement: Document S1. Figures S1–S7 and Tables S1–S3 [file mmc1.pdf]

Molecular Cell, Volume *79*

Supplemental Information

An Insulin-Sensitive Circular RNA

that Regulates Lifespan in *Drosophila*

Carina Marianne Weigelt, Rohan Sehgal, Luke Stephen Tain, Jun Cheng, Jacqueline Eßer, André Pahl, Christoph Dieterich, Sebastian Grönke, and Linda Partridge

Supplemental Figures

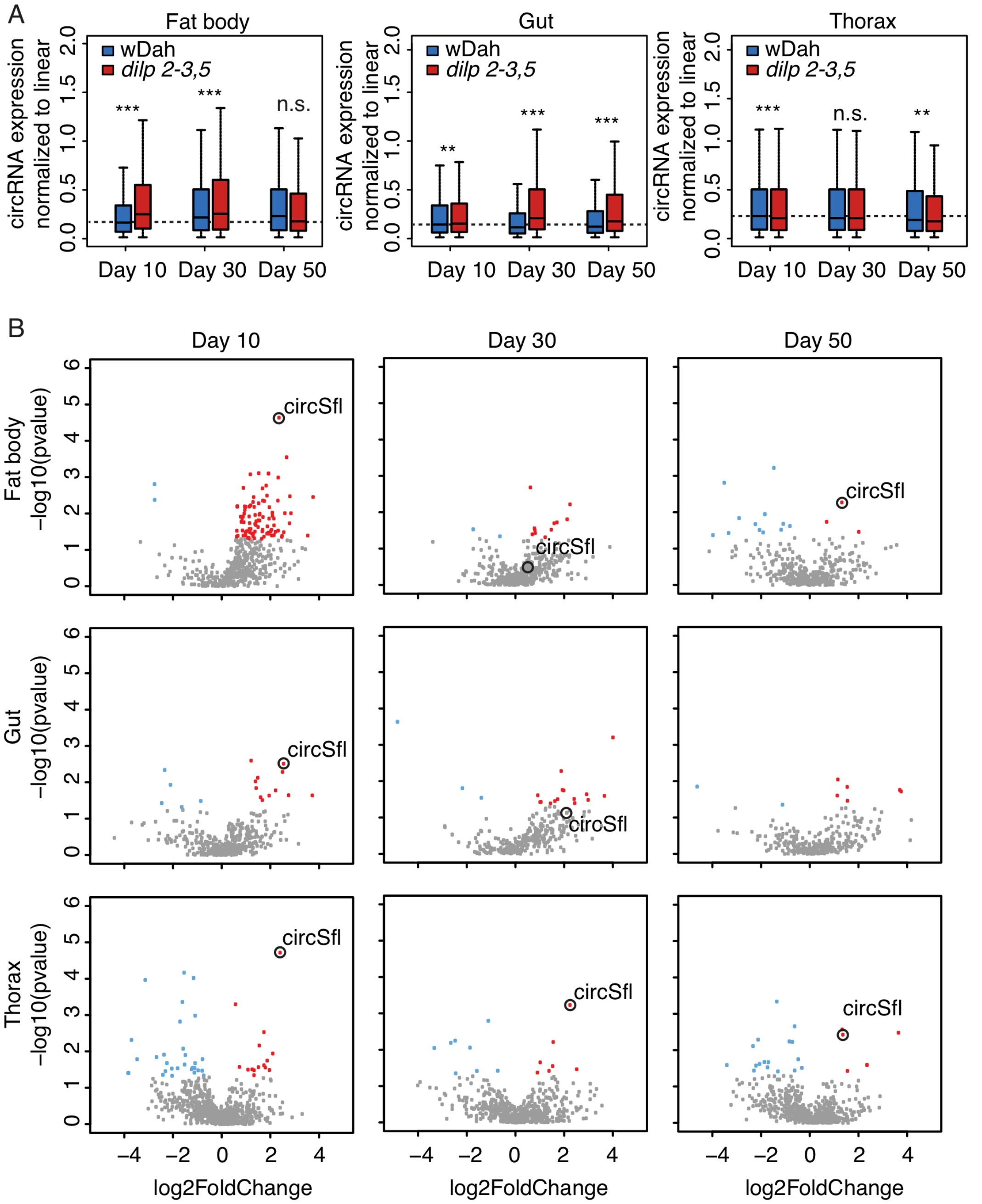

Figure S1: Differentially expressed circRNAs in the fat body, gut and thorax of dilp *2-3,5* mutant flies, Related to Figure 1. (A) Global circRNA expression was only mildly increased during ageing in the fat body, unchanged in the gut and mildly decreased in the thorax of *wDah* wildtype flies. Global circRNA levels were up-regulated in fat body and gut of *dilp 2-3,5* mutants (fat body: genotype $p<0.05$, interaction with age: $p<0.0001$; gut: genotype: $p<0.0001$, interaction with age: $p<0.0001$, 2-way ANOVA, n=3) and not significantly changed in the thorax of *dilp2-3,5* mutants (n=3). (B) CircSfl was up-regulated in most tissues and at all ages in *dilp2-3,5* mutants ($p<0.05$, beta-binomial test, n=3).

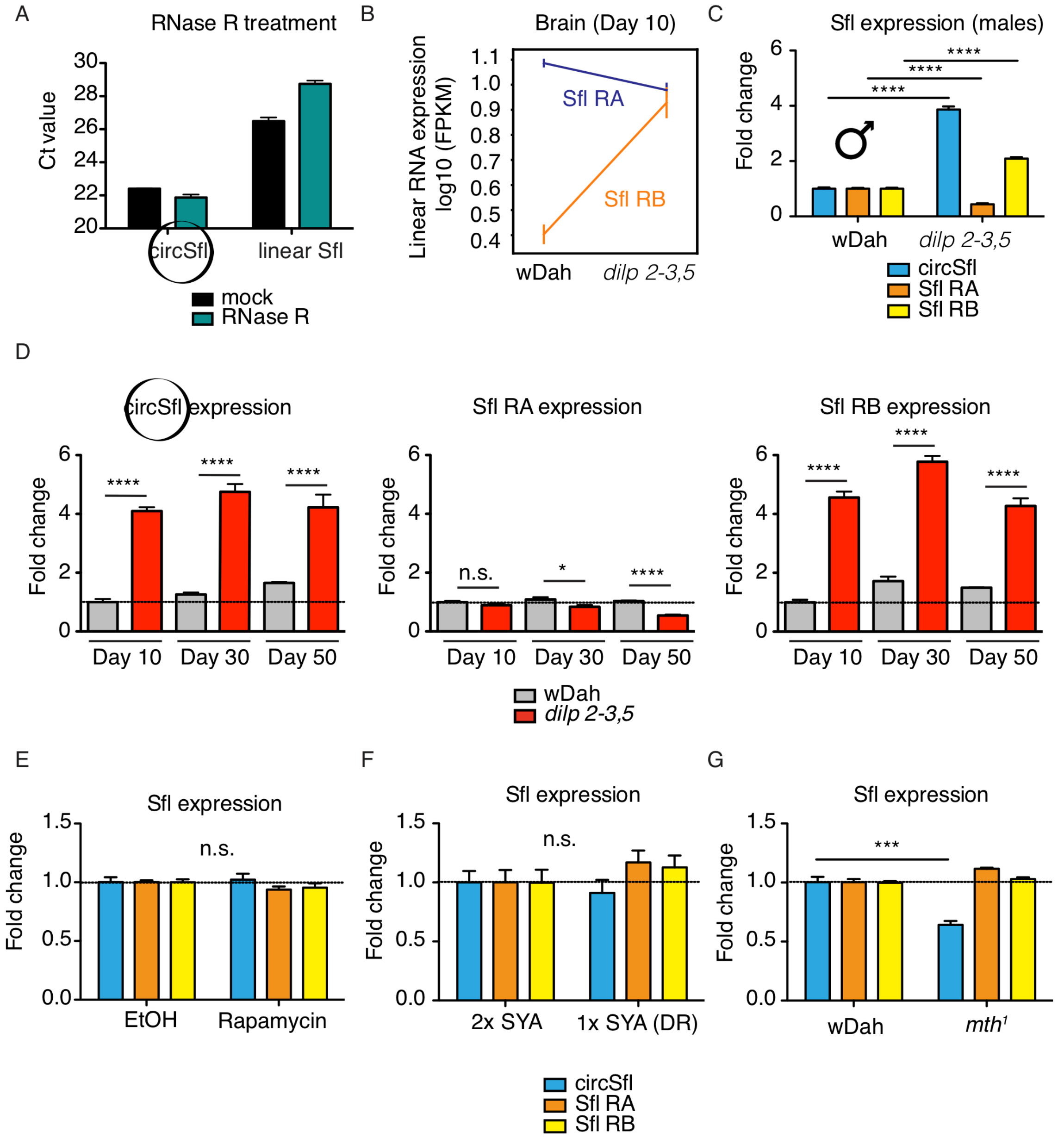

Figure S2: Verification and regulation of circSfl, Related to Figure 2. (A) RNase R treatment did not affect circSfl stability, but degradated linear Sfl transcripts as indicated by higher Ct values as measured by q-RT-PCR and normalized to input. (B) RNA sequencing on brains showed that Sfl RA was the predominantly expressed linear isoform in wildtype flies. In contrast, *dilp 2-3,5* mutants expressed Sfl RA and RB at similar levels. (C) CircSfl was also up-regulated in male *dilp 2-3,5* mutants (whole flies), while Sfl RA was decreased and Sfl RB increased compared to wDah control flies (Student's t-test, n = 4). (D) CircSfl was up-regulated in young, middle-aged and old *dilp2-3,5* mutant fly heads. While Sfl RA transcript levels were not increased in insulin mutant flies, Sfl RB transcript levels were increased at all investigated timepoints (*p<0.05, ***p<0.001, ****p<0.0001, 2-way ANOVA with Bonferroni post-hoc test). (E) Reduction of TOR signalling by application of rapamycin did

not affect circSfl or linear Sfl expression levels in fly heads (Student's t-test, n=4). (F) Dietary restriction (1x SYA) did not affect transcript levels of circSfl, Sfl RA or Sfl RB in comparison to control fully fed female flies (2x SYA) (Student's t-test, n = 4). (G) CircSfl expression was decreased in *mth*[1] mutant female flies (Student's t-test, n = 4).

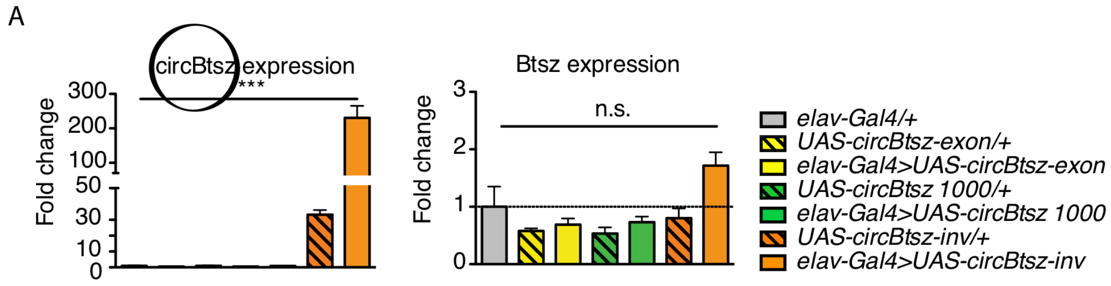

Figure S3: Generation of circBtsz over-expression mutants, Related to Figure 3. (A) CircBtsz was strongly over-expressed in *elav-Gal4>UAS-circBtsz-inv* flies, but not in the other transgenic lines tested (**p<0.01, ***p<0.001, 1-way ANOVA with Tukey post-hoc test, n=3).

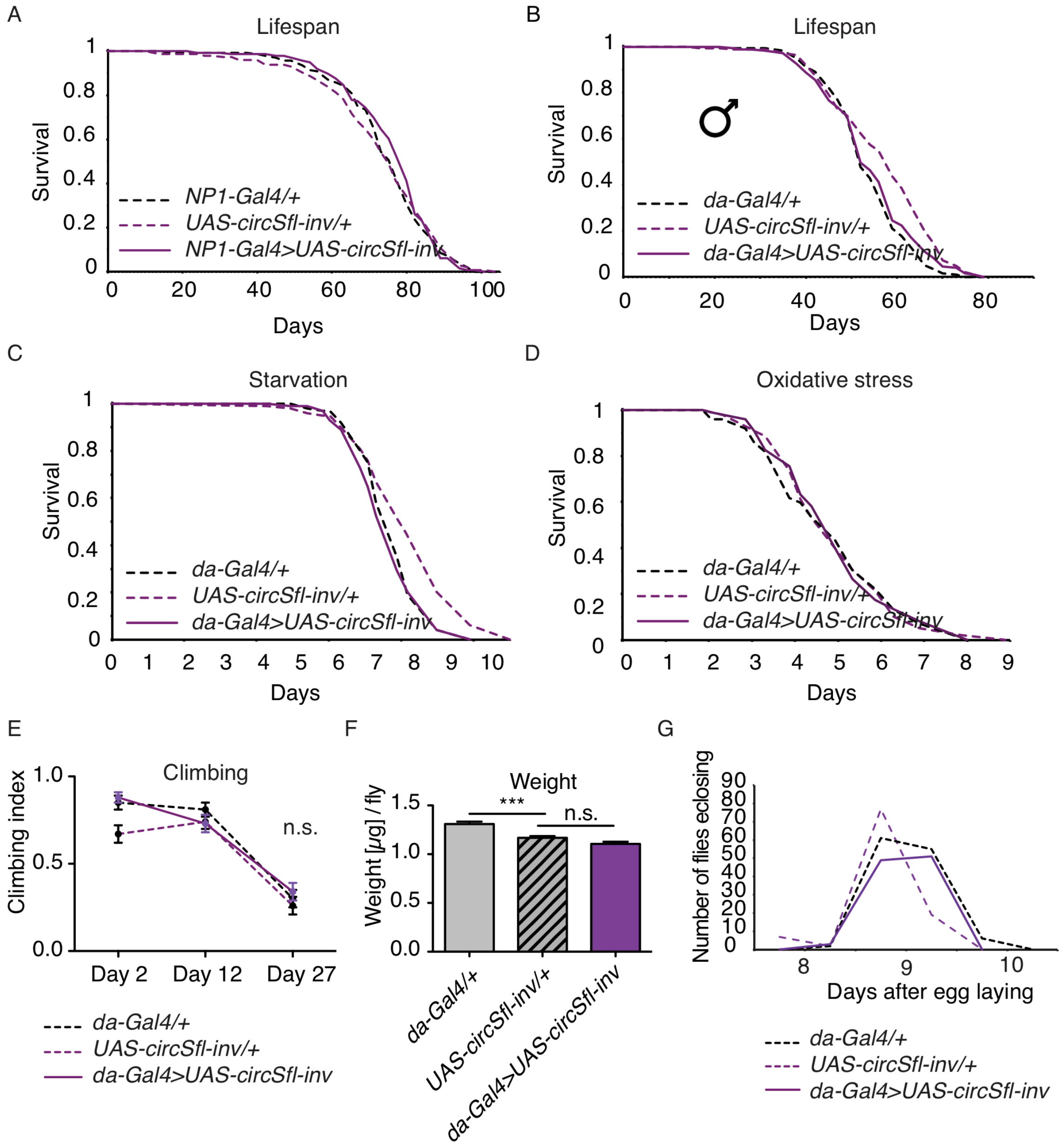

Figure S4: Overexpression of circSfl does not affect stress resistance, body weight or development, Related to Figure 3. (A) Gut-specific over-expression of circSfl by NP1-Gal4 did not affect female lifespan. (B) Ubiquitous over-expression of circSfl did not affect lifespan of male flies. (C-D) CircSfl over-expression mutants showed a normal response to (C) starvation (n~100) and (D) to oxidative stress (log-rank test, n~100). (E) CircSfl over-expression mutants showed normal climbing abilities (2-way ANOVA, n~60). (F) Body weight of circSfl over-expression mutants was not different to the *UAS-circSfl/+* control (1-way ANOVA with Tukey post-hoc test, n=10). (G) Mean development time of circSfl over-expression mutants was not different compared to the *da-Gal4/+* control (1-way ANOVA).

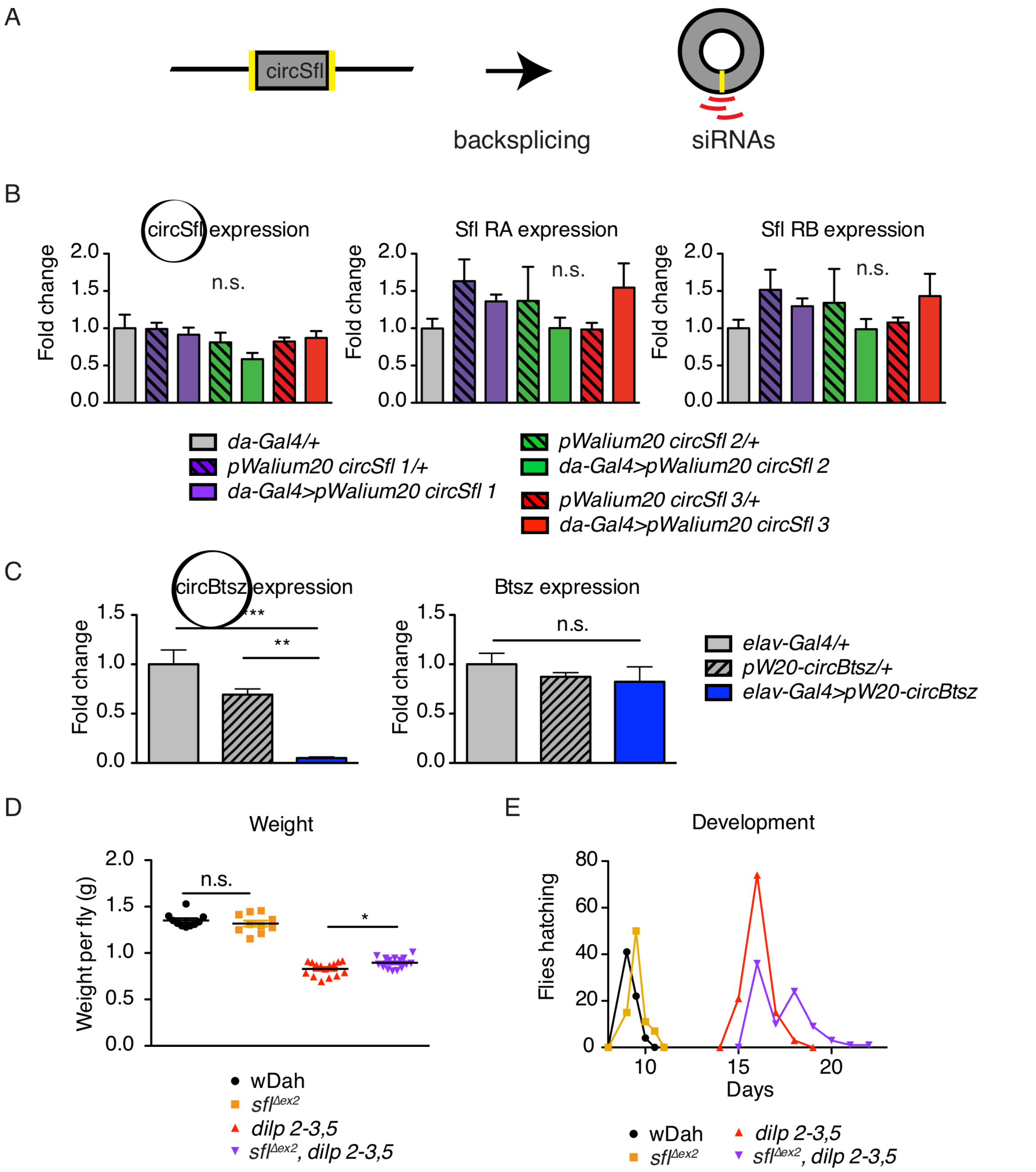

Figure S5: SiRNA mutants to knock-down circRNAs and weight and development of *sfl*$^{\Delta ex2}$ mutants, Related to Figure 4. (A) Three different siRNAs were used to target the circRNA-specific backsplice junction. (B) circSfl expression levels were not significantly decreased in fly lines expressing siRNAs against the circSfl-specific splice junction. (C) CircBtsz expression was strongly reduced in flies expressing siRNAs targeting the circBtsz-specific backsplice junction, but not the linear Btsz transcript. (D) Weight of *sfl*$^{\Delta ex2}$ mutants was similar to wDah control flies. The reduced weight of *dilp 2-3,5* mutants was partially rescued in *sfl*$^{\Delta ex2}$*, dilp 2-3,5* double mutants (*sfl*$^{\Delta ex2}$: n.s., *dilp 2-3,5*: ****p<0.0001, interaction: *p<0.05, 2-way ANOVA with Bonferroni post-hoc test, n = 10-15). (E) *sfl*$^{\Delta ex2}$*, dilp 2-3,5* double mutants were even more developmentally delayed as *dilp 2-3,5* mutants (Mean

eclosion day: *sfl*$^{\Delta ex2}$, *dilp 2-3,5* vs. *dilp 2-3,5:* p<0.0001, interaction between *sfl*$^{\Delta ex2}$ and *dilp 2-3,5: p<0.0001,* 2-way ANOVA with Bonferroni post-hoc test, n ~ 60-130).

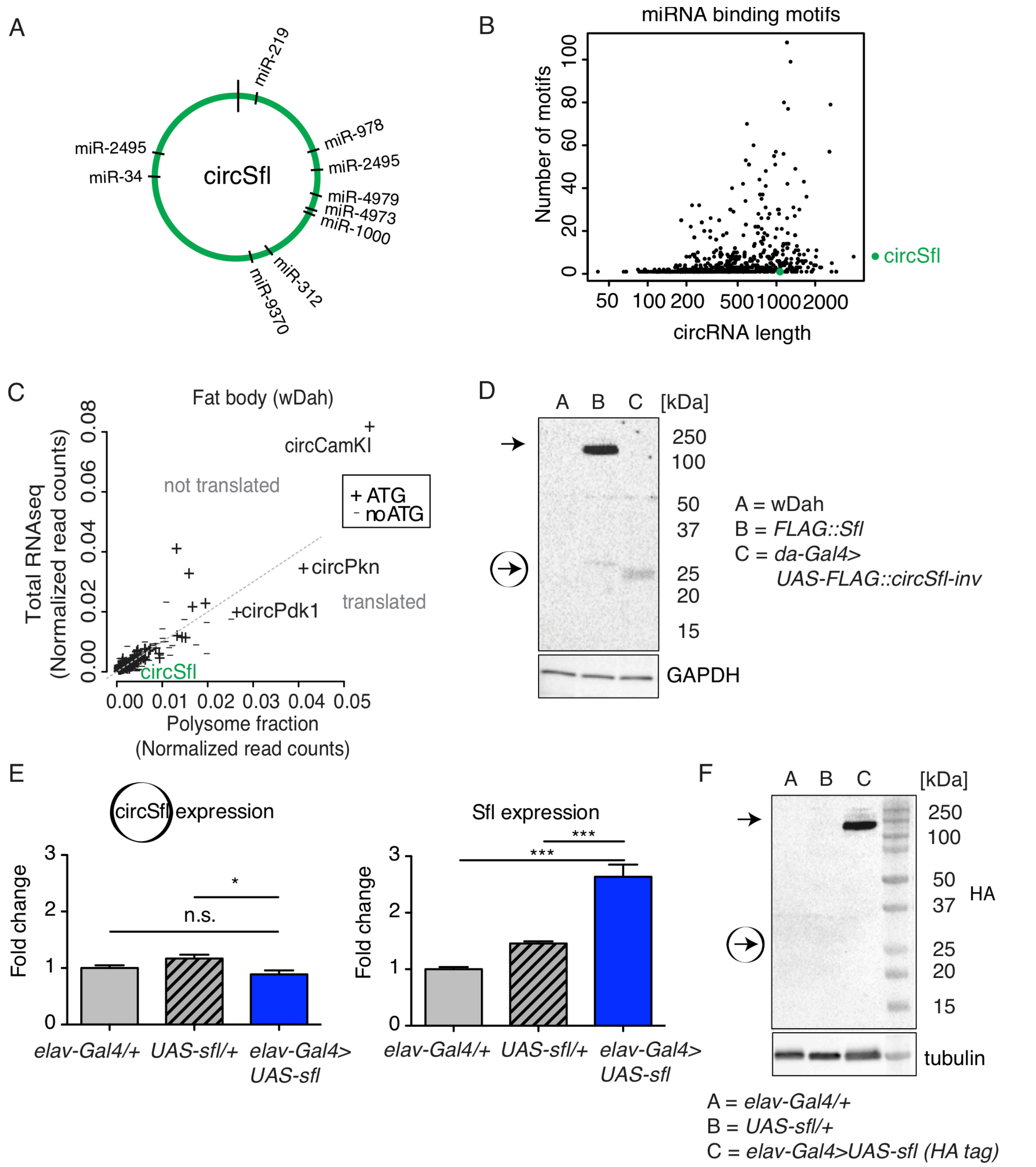

Figure S6: The 25kDa Sfl protein isoform does result from proteolytic cleavage of the full length Sfl protein, Related to Figure 5. (A) 10 different miRNA seed sequences were identified in the circSfl transcript. (B) Number of miRNA binding motifs in circSfl (green) compared to all other circRNAs in *Drosophila*. Note, the number of identified miRNA binding sites in circSfl is lower in this analysis compared to (A), as all circRNs were analysed in parallel, which reduces the power to detect individual sites. (C) Low levels of circSfl were detected by polysome-profiling in wildtype flies indicative for low levels of circSfl translation.

(D) Over-expression of FLAG::circSfl-inv resulted in a single band of around 25 kDa similar to the smaller band observed in *FLAG::Sfl* knock-in flies. (E) Over-expression of an HA-tagged Sfl cDNA *(elav-Gal4>UAS-sfl)* increased linear Sfl transcript levels but not circSfl levels (***p<0.001, *p<0.05, 1-way ANOVA with Tukey post-hoc test, n =3). (F) Western blot analysis identified a single band of around 110 kDa corresponding to the full-length Sfl protein in HA-tag Sfl cDNA over-expressor flies. No protein was detected at the size of 25kDa.

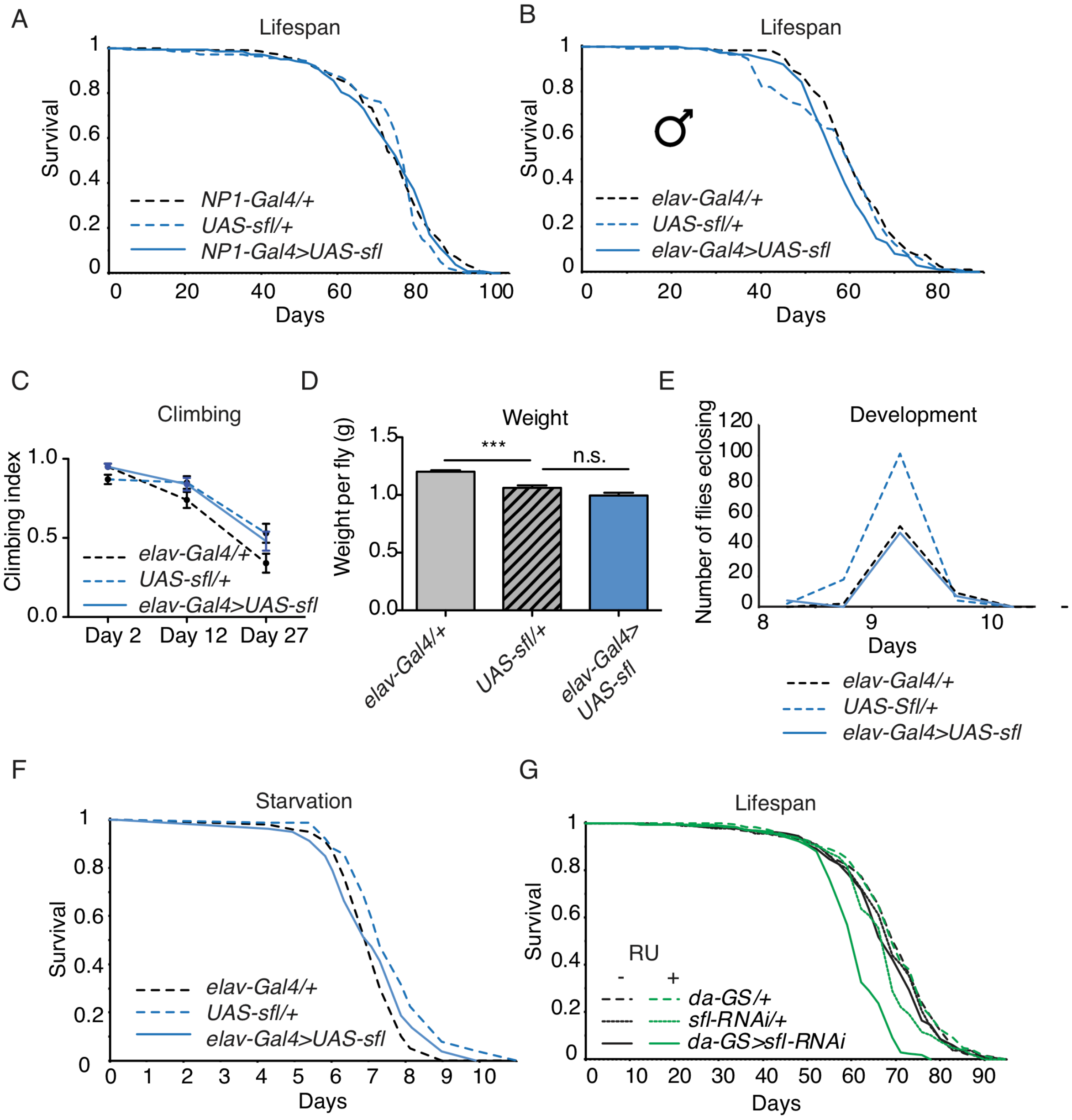

Figure S7: Sfl over-expression mutants have a normal starvation response, climbing ability, weight and development, Related to Figure 6. (A) Over-expression of Sfl in the gut by NP1-Gal4 did not affect lifespan of female flies. (B) Neuron-specific over-expression of

Sfl did not affect lifespan of male flies. (C) Female Sfl over-expression (*elav-Gal4>UAS-sfl*) mutants showed normal climbing ability. (D) The body weight of Sfl over-expression mutants was not different to *UAS-sfl/+* controls (1-way ANOVA with Tukey post-hoc test, n=10). (E) Development time of Sfl over-expression mutants was not changed compared to both controls (1-way ANOVA). (F) Over-expression of Sfl in neurons did not alter starvation resistance. (G) Adult-onset knock-down of Sfl by RU induction shortened lifespan (*da-GS>sfl RNAi* uninduced vs. induced: p<0.0001, log rank test, n~200).

Supplemental Tables

Table S1: Cloning strategy for vectors generated in this study including primers used., Related to STAR Methods. Underlined bases highlight the restriction site used. **Fat** bases highlight siRNA or guideRNA sequences within the primers.

Vector name	Vector backbone	Primer names	Primer sequences	Method
pUAST attb-circBtsz-exon	pUAST attb	CW113	ATGAATTCGTATCAATTTGCCCACCGGCAGG	Restriction digest via EcoRI and NotI (EcoRI is naturally present)
		CW114	ATGCGGCCGCATCATTCTAGTGGCATCTCTCTGCC	
pUAST attb circBtsz-1000	pUAST attb	CW102	AGTGAATTCTCATTGAATTGGCTTTGGA	Restriction digest via EcoRI and NotI (EcoRI is naturally present)
		CW103	ATGCGGCCGCGGCAACAATTTTCCGCGCATATCA	
pUAST attb circBtsz-inv	pUAST attb	CW102	AGTGAATTCTCATTGAATTGGCTTTGGA	1) Restriction digest via EcoRI + NotI (CW102 + 192)<br>2) Restriction digest via NotI (CW193 + 194)
		CW192	ATGCGGCCGCCGTTCGGTCGATATATGTGGCTCTAA	
		CW193	ATGCGGCCGCTCATTGAATTGGCTTTGGA	
		CW194	ATGCGGCCGCGCCCCTACAATTCCCTGTTCATATTT	
pWalium20 circBtsz-1	pWalium 20	CW168	CTAGCAGT**CACTAGAATGATGTATCAATT**TAGTTATATTCAAGCATA**AATTGATACATCATTCTAGTG**GCG	pWalium cloning<br><br>Ni et al. (2008)
		CW169	AATTCGC**CACTAGAATGATGTATCAATT**TATGCTTGAATATAACTA**AATTGATACATCATTCTAGTG**ACTG	
		CW187	ATGAGCTCTTTAATTAGGAAAGCAAGGGAGAGGCGG	
pUAST	pUAST	CW090	ATGCGGCCGCATAAGAGG	Restriction digest

attb-circSfl-exon	attb		ACGACCCACTGGGACG	with NotI and KpnI
		CW091	ATGGTACCCCATTAAACACGCCCGTATCGACAT	
pUAST attb-circSfl-1000	pUAST attb	CW085	ATGCGGCCGCCAAACATTGTTGGCCATCTAAAACT	Restriction digest via NotI and KpnI
		CW086	ATGGTACCGCTTGCACCAAATGAGTTTTATTCT	
pUAST attb-circSfl-inv	pUAST attb	CW085	ATGCGGCCGCCAAACATTGTTGGCCATCTAAAACT	1) Restriction digest via NotI and KpnI (CW085 + 088)<br>2) Restriction digest via KpnI (CW087 + CW089)
		CW088	ATGGTACCTGTCTCCCACATAGTTTCAAATCCCA	
		CW087	ATGGTACCCAAACATTGTTGGCCATCTAAAACT	
		CW089	ATGGTACCTCTAATCAGGTCAAAAGTTGTTAGCCT	
pUAST attb FLAG::circSfl-inv	pUAST attb-circSfl-inv	Gene Synthesis	Add FLAG tag before circSfl ORF	1) Restriction digest via NotI and KpnI<br>2) Subclone in pBS SK<br>3) Insert GeneSynthesis product via XbaI + NsiI<br>4) Clone into pUAST attb via NotI + KpnI<br>5) Add intron via KpnI
pCFD4 Sfl N Term	pCFD4	CW224	TATATAGGAAAGATATCCGGGTGAACTTC**GTCGCTCTGTTTAGTCAATAT**GTTTTAGAGCTAGAAATAGCAAG	pCFD4 Cloning<br><br>Port et al. (2014)
		CW225	ATTTTAACTTGCTATTTCTAGCTCTAAAAC**TGACCGGCACCCAAACGGAT**CGACGTTAAATTGAAAATAGGTC	
pBS FLAG::Sfl Donor	pBluescript SK+	CW265	ATGGTACCACCGAAATGCCACCATCAATTAGTT	1) Restriction Digest via KpnI + NotI<br>2) Insert Gene Synthesis Product via EcoRI and HindIII
		CW266	ATGCGGCCGCAAACCGAGCAGACTGAAGTGCATTT	
pWalium20-circSfl-1	pWalium 20	CW164	CTAGCAGT**GTGTTTAATGGATAAGAGGAC**TAGTTATATTCAAGCATA**GTCCTCTTATCCATTAAACAC**GCG	pWalium cloning<br><br>Ni et al. (2008)
		CW165	AATTCGC**GTGTTTAATGGATAAGAGGAC**TATGCTTGAATATAACTA**GTCCTCTTATCCATTAAACAC**ACTG	
pWalium20-circSfl-2	pWalium 20	CW360	CTAGCAGT**GCGTGTTTAATGGATAAGAGG**TAGTTATATTCAAGCATA**CCTCTTA**	pWalium cloning<br><br>Ni et al. (2008)

			**TCCATTAAACACGC**GCG	
		CW361	AATTCGC**GCGTGTTTAATGGATAAGAGG**TATGCTTGAATATAACTA**CCTCTTATCCATTAAACACGC**ACTG	
pWalium20-circSfl-3	pWalium 20	CW362	CTAGCAGT**GTTTAATGGATAAGAGGACGA**TAGTTATATTCAAGCATA**TCGTCCTCTTATCCATTAAAC**GCG	pWalium cloning<br><br>Ni et al. (2008)
		CW363	AATTCGC**GTTTAATGGATAAGAGGACGA**TATGCTTGAATATAACTA**TCGTCCTCTTATCCATTAAAC**ACTG	
pCFD4 $sfl^{\Delta ex2}$	pCFD4	CW184	TATATAGGAAAGATATCCGGGTGAACTTC**GACCAGCTTGCGGAGATTCGC**GTTTTAGAGCTAGAAATAGCAAG	pCFD4 Cloning<br><br>Port et al. (2014)
		CW185	ATTTTAACTTGCTATTTCTAGCTCTAAAAC**AATTTTATCCAGTAATGAAAC**GACGTTAAATTGAAAATAGGTC	
pUAST-attb-circSfl-ORF	pUAST attb	CW273	ATGCGGCCGCATGACAATATCGGGCGGCAATCA	Restriction digest via NotI + KpnI
		CW274	ATGGTACCTTATCCATTAACACGCCCGTATCG	

Table S2: Transgenic flies generated in this study, Related to STAR Methods.

Transgenic fly line	Vector used	Injected into
UAS-circBtsz-exon	pUAST attb circBtsz-exon	y^1w^1; vas-phiC31; attP40
UAS-circBtsz-1000	pUAST-circBtsz-1000	y^1w^1; vas-phiC31; attP40
UAS-circBtsz-inv	pUAST-circBtsz-inv	y^1w^1; vas-phiC31; attP40
pWalium20-circBtsz-1	pWalium20-circBtsz-1	y^1w^1; vas-phiC31; attP40
UAS-circSfl-exon	pUAST attb circSfl-exon	y^1w^1; vas-phiC31; attP40
UAS-circSfl-1000	pUAST attb circSfl-1000	y^1w^1; vas-phiC31; attP40
UAS-circSfl-inv	pUAST attb circSfl-inv	y^1w^1; vas-phiC31; attP40
UAS-FLAG::circSfl-inv	pUAST attb FLAG::circSfl-inv	y^1w^1; vas-phiC31; attP40
pCFD4 Sfl N term	pCFD4 Sfl N term	y^1v^1; nanos-phiC31; attP40
FLAG::Sfl	pBS FLAG::Sfl	Progeny of nos-Cas9 x pCFD4 Sfl N term
pWalium20-circSfl-1	pWalium20-circSfl-1	y^1w^1; vas-phiC31; attP40
pWalium20-circSfl-2	pWalium20-circSfl-2	y^1w^1; vas-phiC31; attP40
pWalium20-circSfl-3	pWalium20-circSfl-3	y^1w^1; vas-phiC31; attP40

pCFD4 sfl$^{\Delta ex2}$	pCFD4 sfl$^{\Delta ex2}$	y^{1}v^1; nanos-phiC31; attP40
sfl$^{\Delta ex2}$	-	Progeny of nos-Cas9 x pCFD4 sfl$^{\Delta ex2}$
UAS-circSfl-ORF	pUAST attb circSfl-ORF	y^{1}w^1; vas-phiC31; attP40

Table S3: Oligonucleotides used for q-RT-PCR in this study, Related to STAR Methods.

Target gene	Forward primer	Sequence (for)	Reverse primer	Sequence (rev)
Rpl32	SOL268	ATATGCTAAGCTGTCGCACAAATGG	SOL269	GATCCGTAACCGATGTTGGGCA
circSfl	CW061	ATGTCGATACGGGCGTGTTT	CW062	CCAGACTGTCCACTCGCAAT
Sfl RA	CW170	ATTGAAGCTGTCGATTTGAGT	CW062	CCAGACTGTCCACTCGCAAT
Sfl RB	CW171	TGTGCGACAGATAAGAGGACG	CW062	CCAGACTGTCCACTCGCAAT
Sfl all	CW078	TCCCACGGCGATGTTATAGC	CW079	CAGATCCTTCAGTGCCCTCG
circ-Btsz	CW049	AGTCGGTGAGGCAGAGAGAT	CW050	CTGCTTTGCGGCCGAAATG
Btsz	CW049	AGTCGGTGAGGCAGAGAGAT	CW071	GGTTGTTGTGTGGCATCGAG
