# Supplementary material for: An Insulin-Sensitive Circular RNA that Regulates Lifespan in Drosophila
Source: Mol Cell. 2020 Jul 16;79(2):268–279.e5. doi: 10.1016/j.molcel.2020.06.011 (PMC7318944; doi:10.1016/j.molcel.2020.06.011)
Supplement: Document S2. Article plus Supplemental Information [file mmc5.pdf]

Molecular Cell

Article

An Insulin-Sensitive Circular RNA that Regulates Lifespan in *Drosophila*

Graphical Abstract

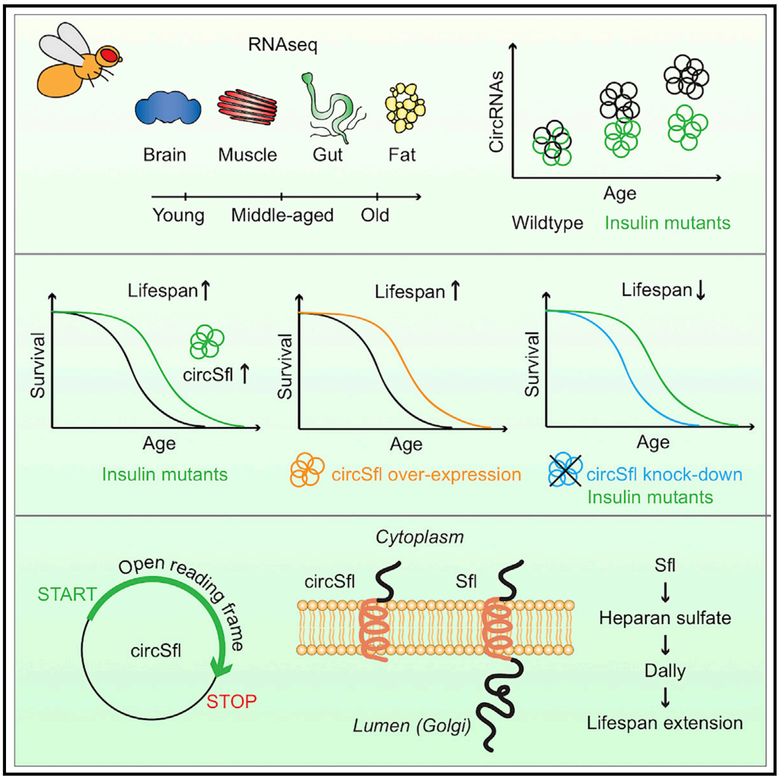

Highlights

- Accumulation of circRNAs with age is slowed down in long-lived insulin mutant flies
- A circRNA encoded by the *sulfateless* gene is induced in long-lived insulin mutants
- Overexpression of circSfl extends the lifespan of fruit flies
- CircSfl is translated, and the resulting peptide is sufficient to extend lifespan

Authors

Carina Marianne Weigelt,
Rohan Sehgal, Luke Stephen Tain, ...,
Christoph Dieterich, Sebastian Grönke,
Linda Partridge

Correspondence

sgroenke@age.mpg.de (S.G.),
partridge@age.mpg.de (L.P.)

In Brief

Circular RNAs are a poorly characterized class of non-coding RNAs that accumulate with age in diverse species. In this study, Weigelt et al. identify a circular RNA that is specifically regulated in long-lived insulin mutant flies and extends the lifespan of fruit flies.

Weigelt et al., 2020, Molecular Cell *79*, 268–279
July 16, 2020 © 2020 The Authors. Published by Elsevier Inc.
https://doi.org/10.1016/j.molcel.2020.06.011

CellPress

CellPress
OPEN ACCESS

Molecular Cell

Article

An Insulin-Sensitive Circular RNA that Regulates Lifespan in *Drosophila*

Carina Marianne Weigelt,[1] Rohan Sehgal,[1] Luke Stephen Tain,[1] Jun Cheng,[1] Jacqueline Eßer,[1] André Pahl,[1] Christoph Dieterich,[1,2] Sebastian Grönke,[1,*] and Linda Partridge[1,3,4,*]
[1]Max Planck Institute for Biology of Ageing, Joseph-Stelzmann-Str. 9b, 50931 Cologne, Germany
[2]Section of Bioinformatics and Systems Cardiology, University Hospital Heidelberg, 69120 Heidelberg, Germany
[3]Institute of Healthy Ageing, Genetics, Evolution and Environment, University College London, Darwin Building, Gower Street, London WC1E 6BT, UK
[4]Lead Contact
*Correspondence: sgroenke@age.mpg.de (S.G.), partridge@age.mpg.de (L.P.)
https://doi.org/10.1016/j.molcel.2020.06.011

SUMMARY

Circular RNAs (circRNAs) are abundant and accumulate with age in neurons of diverse species. However, only few circRNAs have been functionally characterized, and their role during aging has not been addressed. Here, we use transcriptome profiling during aging and find that accumulation of circRNAs is slowed down in long-lived insulin mutant flies. Next, we characterize the *in vivo* function of a circRNA generated by the *sulfateless* gene (circSfl), which is consistently upregulated, particularly in the brain and muscle, of diverse long-lived insulin mutants. Strikingly, lifespan extension of insulin mutants is dependent on circSfl, and overexpression of circSfl alone is sufficient to extend the lifespan. Moreover, circSfl is translated into a protein that shares the N terminus and potentially some functions with the full-length Sfl protein encoded by the host gene. Our study demonstrates that insulin signaling affects global circRNA accumulation and reveals an important role of circSfl during aging *in vivo*.

INTRODUCTION

Circular RNAs (circRNAs) were originally identified more than 30 years ago, but for a long time they were thought to be by-products of the mRNA splicing process without a specific function; hence, they were not investigated further (Grabowski et al., 1981; Capel et al., 1993; Nigro et al., 1991; Cocquerelle et al., 1993). Recently, circRNAs have been discovered in fungi, protists, and plants (Wang et al., 2014); *C. elegans* (Memczak et al., 2013); *Drosophila* (Salzman et al., 2013); mice (Jeck et al., 2013); and humans (Salzman et al., 2012). The majority of circRNA are generated by backsplicing of exons of protein-coding genes (“host genes”) (Figure 1A), and reverse complementary regions in the introns flanking circRNA-producing exons are crucial for circularization (Ashwal-Fluss et al., 2014; Ivanov et al., 2015; Starke et al., 2015). Despite the high abundance and expression of certain circRNAs (Salzman et al., 2012), only a few circRNAs have been functionally characterized; for instance, human CDR1as, which acts as an effective microRNA sponge (Kleaveland et al., 2018; Piwecka et al., 2017; Memczak et al., 2013; Hansen et al., 2013). More recently, two independent reports have shown that a subset of circRNAs might be translated (Legnini et al., 2017; Pamudurti et al., 2017). circRNAs are enriched in neuronal tissues such as *Drosophila* heads (Westholm et al., 2014) and the mammalian brain (Rybak-Wolf et al., 2015). Furthermore, circRNAs have been shown to accumulate with age in *C. elegans* (Cortés-López et al., 2018), in *Drosophila* heads and photoreceptor neurons (Westholm et al., 2014; Hall et al., 2017), and in the mouse cortex and hippocampus but not in mouse heart tissue (Gruner et al., 2016). However, a function of circRNAs in the aging process has not yet been revealed.

The nutrient-sensing insulin/insulin-like growth factor signaling (IIS) pathway is a key regulator of aging, metabolism, reproduction, and growth and is evolutionarily conserved from worms and flies to mice and humans. Downregulation of IIS pathway activity pharmacologically or by genetic modification extends the lifespan in *C. elegans* (Kenyon et al., 1993), *Drosophila* (Clancy et al., 2001), and mice (Selman et al., 2008). In *Drosophila*, simultaneous knockout of three of the seven insulin-like peptides (*dilp2-3,5*) results in a robust lifespan extension of 30%–50% (Grönke et al., 2010) and ameliorates the age-related decline in sleep quality (Metaxakis et al., 2014), suggesting that the healthspan is also extended. Proteome analysis of long-lived insulin mutants (genetic ablation of insulin-producing cells) revealed that the response to reduced insulin signaling and lifespan extension are highly tissue specific (Tain et al., 2017).

In this study, we characterized the functional link between circRNAs and insulin-mediated lifespan extension and aging.

This is an open access article under the CC BY license (http://creativecommons.org/licenses/by/4.0/).

CellPress
OPEN ACCESS

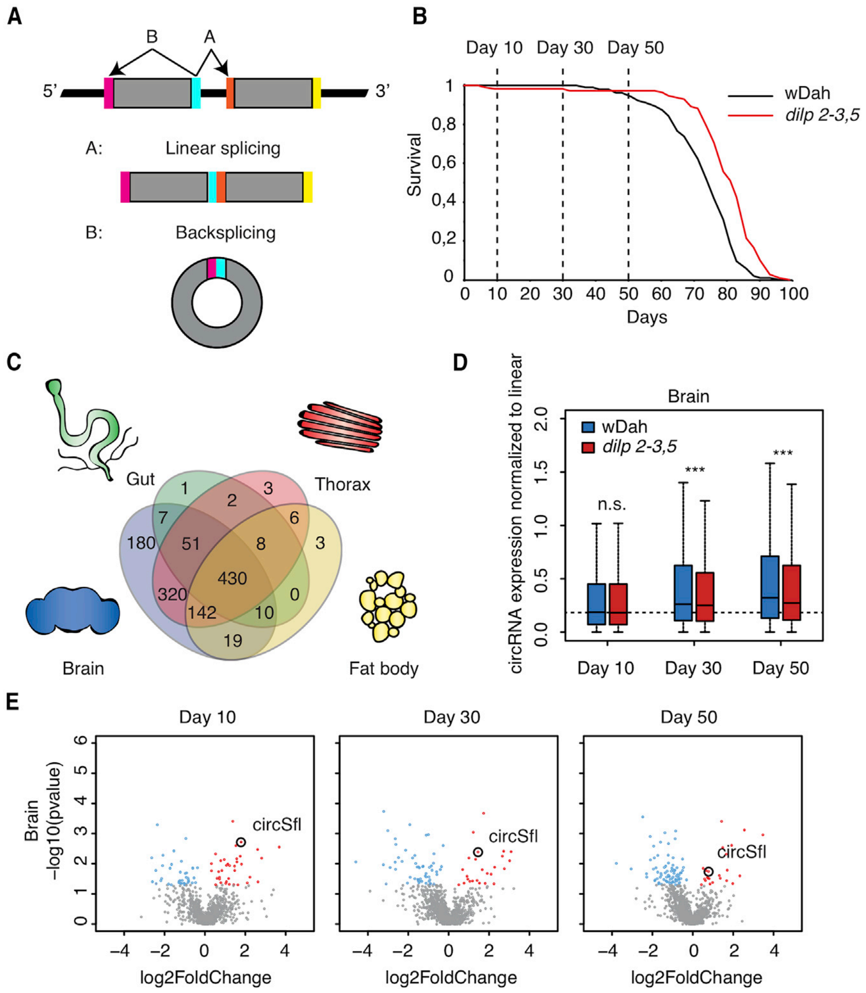

Figure 1. Tissue-Specific circRNA Profiling in Long-Lived Insulin Mutant Flies during Aging

(A) Schematic overview of circRNA biogenesis by backsplicing.

(B) For circRNA profiling, tissues of wild-type wDah flies and long-lived *dilp 2-3,5* mutants were collected from young (day 10), middle-aged (day 30), and old (day 50) female flies.

(C) circRNAs were highly enriched in the brain of wDah control flies compared with the thorax, gut, and fat body.

(D) Global accumulation of circRNAs in the brain with age was reduced in long-lived *dilp 2-3,5* mutant flies (age, $p < 0.0001$; genotype, $p < 0.001$; interaction, $p < 0.05$; 2-way ANOVA, $n = 3$, median with 25th/75th percentile [box] and minimum/maximum [error bars]).

(E) Volcano plots of differentially expressed circRNAs in brains of *dilp 2-3,5* mutant flies at days 10, 30, and 50. Significantly upregulated circRNAs are highlighted in red and significantly downregulated circRNAs in blue ($p < 0.05$, beta-binomial test, $n = 3$). CircRNA expression was normalized to its host gene. circSfl was consistently upregulated in *dilp 2-3,5* mutant flies.

See also Figure S1 and Data S1 and S2.

We used tissue-specific, genome-wide, next-generation sequencing of wild-type and *dilp2-3,5* mutant flies and identified hundreds of differentially expressed circRNAs, including the circRNA encoded by the *sulfateless* (*sfl*) gene (hereafter referred to as circSfl). circSfl was highly upregulated in all tissues of several long-lived insulin mutants, and overexpression of circSfl alone was sufficient to extend the lifespan. Finally, we provide evidence that circSfl is translated into a small protein that may share some function with the protein encoded by the linear *sfl* transcripts. Importantly, overexpression of just the circSfl open reading frame (ORF) from a linear transcript was sufficient to extend longevity, implicating the protein encoded by circSfl in lifespan regulation. We demonstrated that circRNAs are actively involved in the aging process and can influence the lifespan.

RESULTS

circRNA Accumulation with Age in Neuronal Tissue Is Slowed Down in Insulin Mutants

circRNAs accumulate with age in various organisms (Cortés-López et al., 2018; Gruner et al., 2016; Westholm et al., 2014; Hall et al., 2017). However, the function of circRNAs *in vivo* during aging is still elusive. To study the dynamics of circRNA expression with age, we conducted transcriptome-wide deep sequencing of wild-type (wDah) *Drosophila* flies and long-lived *dilp 2-3,5* mutants (Figure 1B). We dissected the four main adult fly tissues (brain, gut with malpighian tubules, thorax, and fat body) in young (day 10), middle-aged (day 30), and old (day 50) flies to study the aging process in a tissue-specific manner. Using sequencing reads that spanned the circRNA-specific backsplice junction to detect and quantify circRNAs by DCC (Cheng et al., 2016), we identified, in total, 1,182 circRNAs in wild-type flies (Data S1). In line with previous publications (Westholm et al., 2014), we found a strong bias for circRNAs to be expressed in the brain of wild-type flies (Figure 1C). 1,159 of 1,182 circRNAs were detected in the brain, and 180 of these were brain-specific and not expressed in any other tissue. In contrast, only a few circRNAs were specifically expressed in the gut, thorax, or fat body. Next, we looked at global changes in circRNA expression levels with age relative to their host genes. As expected, global circRNA expression increased with age in brain tissue (Figure 1D), in contrast to the other tissues, where we observed only very mild accumulation (fat body), no global changes (gut), or slightly reduced circRNA levels (thorax) (Figure S1A). Strikingly, circRNAs also accumulated in *dilp 2-3,5* mutant brains but to a significantly lower extent than in wild-type flies (interaction: $^{*}p < 0.05$, 2-way ANOVA) (Figure 1D), suggesting that circRNA accumulation with age is slowed down by reduced insulin signaling. We noticed that lowered insulin signaling also affected global circRNA level at various time points in the other tissues; e.g., increased circRNA levels in

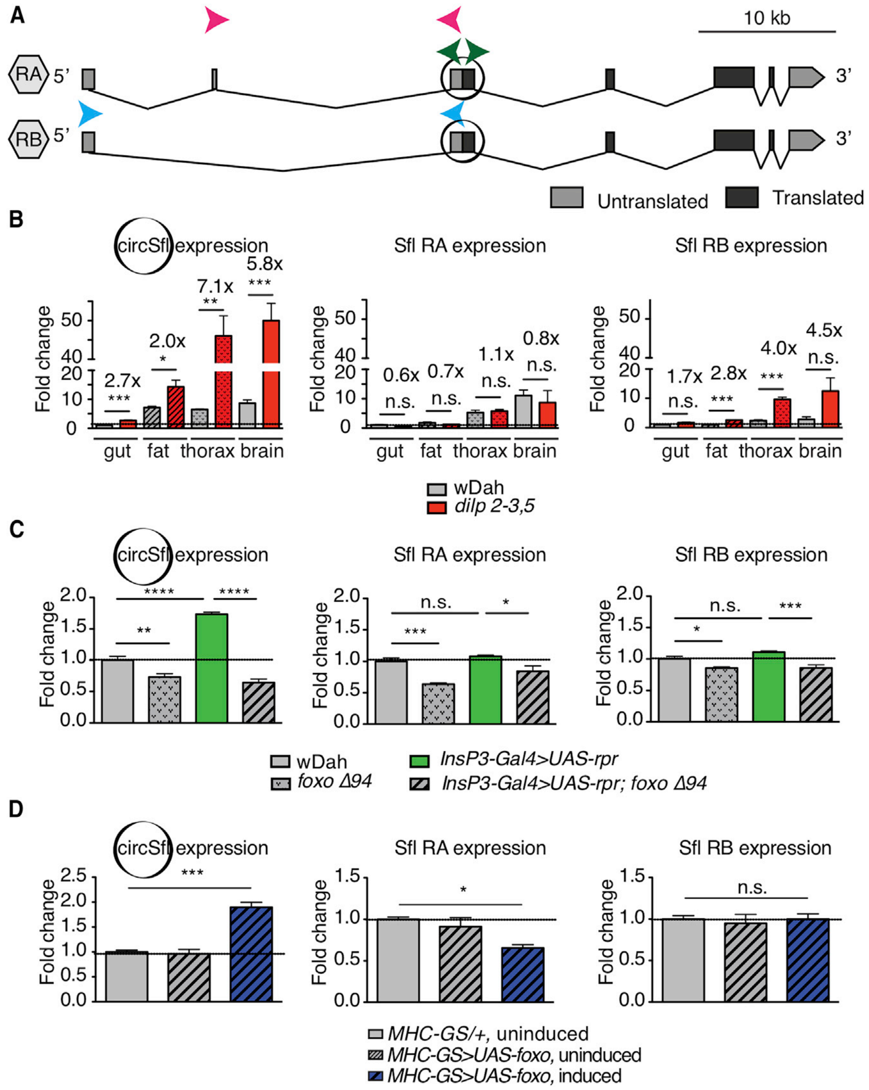

Figure 2. CircSfl Is Upregulated in Long-Lived Insulin Mutant Flies in a *dFoxo*-Dependent Manner

(A) Scheme of the *sulfateless* gene locus, including primers used to differentiate between circSfl (green arrows), Sfl RA (pink arrows), and Sfl RB (blue arrows).

(B) circSfl and Sfl RB, but not Sfl RA, were upregulated in all tissues of *dilp 2-3,5* mutants (*p < 0.05, **p < 0.01, ***p < 0.001, Student's t test, mean ± SEM).

(C) circSfl, but not Sfl RA and RB, was upregulated in flies lacking insulin-producing cells (*InsP3>UAS-rpr*, whole female flies). Upregulation of circSfl was dependent on the *dFoxo* transcription factor (**p < 0.01, ****p < 0.0001; interaction MNC ablation and dFoxo, p < 0.0001; 2-way ANOVA with Bonferroni post hoc test, n = 3, mean ± SEM).

(D) circSfl was upregulated in thoraces of flies overexpressing dFoxo in muscle (***p < 0.001, 1-way ANOVA, n = 3, mean ± SEM), Sfl RA was downregulated, and expression of Sfl RB was unchanged. All flies were 10 days old.

See also Figure S2.

dilp 2-3,5 gut samples and decreased circRNA levels in *dilp 2-3,5* thoraces but no consistent changes during aging (Figure S1A). In summary, we observed striking differences in circRNA expression not only in a tissue-specific but also in a time-specific manner, supporting the importance of studying the role of circRNAs in the aging process.

CircSfl Is Upregulated in Different Long-Lived Insulin Mutant Flies

Next, we looked at individual circRNAs that were differentially regulated in *dilp 2-3,5* mutants independent of the host gene (Data S2) because this would suggest that regulation of the circRNA is specific and not merely co-regulation with the host gene. The majority of differentially expressed circRNAs were detected in the brain and fat body of *dilp 2-3,5* mutant flies (Figures 1E and S1B), with only a few circRNAs differentially regulated in *dilp 2-3,5* gut and thorax tissues. We identified a circRNA encoded by the *sulfateless* (*sfl*) gene as a prime target for *in vivo* characterization because circSfl was ubiquitously expressed and upregulated in almost all tissues and at almost all time points and was one of the most strongly regulated circRNAs. We verified that circSfl is indeed circular by RNase R treatment, which digests linear RNA species but not circular transcripts (Suzuki et al., 2006). As expected, RNase R digested the linear transcripts of *sfl*, but not circSfl (Figure S2A). Furthermore, Sanger sequencing verified the presence of the circRNA-specific back-splice junction (data not shown), demonstrating that circSfl is indeed a circular transcript. To verify our RNA sequencing data, we analyzed circSfl and linear transcripts from the *sfl* gene by qRT-PCR using specific primers to detect circSfl and the two different linear isoforms RA and RB, differing in a 37-bp long exon with unknown function in the 5′ untranslated region (UTR) of the *sfl* locus (Figure 2A). Our RNA sequencing data indicated that, in wild-type flies, Sfl RA is the predominant isoform and up to 5-fold more highly expressed than Sfl RB (Figure S2B), depending on the tissue and time point. In line with our previous results, circSfl expression was increased up to 7-fold in all tissues of long-lived *dilp 2-3,5* female flies (Figure 2B). Interestingly, expression of the Sfl RB linear isoform, but not the RA isoform, correlated with the circSfl expression pattern in *dilp 2-3,5* mutants (Figure 2B), suggesting a link between splicing of the linear and circular isoforms. Furthermore, consistent upregulation of circSfl in the *dilp 2-3,5* mutant was also observed in male flies (Figure S2C), as well as in the heads of young, middle-aged, and old female flies (Figure S2D). In line with upregulation of circSfl in *dilp 2-3,5* mutants, circSfl expression was also upregulated in other long-lived insulin mutants: those with genetic ablation of median neurosecretory cells (MNCs), which produce insulin (Figure 2C; Broughton et al.,

CellPress
OPEN ACCESS

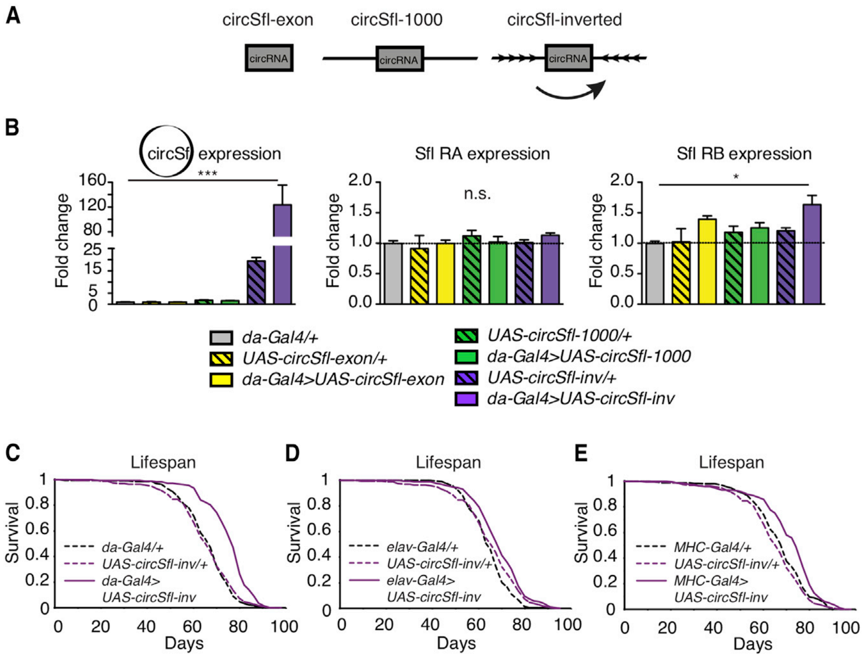

Figure 3. Overexpression of circSfl Extends the Lifespan

(A) Three different overexpression constructs were designed to express circSfl *in vivo*: circSfl-exon (exon only), circSfl-1,000 (circSfl exon + 1,000-bp flanking introns upstream and downstream), and circSfl-inverted (circSfl-inv; circSfl exon + reverse complementary, inverted upstream intron).

(B) circSfl expression was strongly upregulated in flies expressing circSfl-inv (*p < 0.05, ***p < 0.001, 1-way ANOVA, mean ± SEM) but not in circSfl-exon- or circSfl-1,000-expressing flies. The linear transcripts RA and RB of *sfl* were largely unaffected by expression of circSfl constructs.

(C–E) The lifespan of female flies was extended significantly by overexpression of circSfl using the ubiquitous *da-Gal4* driver (C) (overexpressor versus driver or UAS control, ****p < 0.0001), the neuron-specific *elav-Gal4* driver (D) (overexpressor versus driver control, ****p < 0.0001; overexpressor versus UAS control, p < 0.05), and the muscle-specific *MHC-Gal4* driver (E) (overexpressor versus driver control, p < 0.001; overexpressor versus UAS control, ****p < 0.0001) (log rank test, n = ~ 200).

See also Figures S3 and S4.

2005), or overexpression of *dFoxo* in muscles (Figure 2D; Demontis and Perrimon, 2010). In both cases, expression of circSfl, but not of the linear transcripts Sfl RA and RB, was upregulated. Interestingly, circSfl upregulation in MNC-ablated flies was also dependent on dFoxo (2-way ANOVA interaction term, p < 0.0001; Figure 2C), a downstream transcription factor of insulin signaling required for lifespan extension in MNC-ablated flies (Slack et al., 2011). Notably, circSfl expression was not increased in flies fed the lifespan-extending drug rapamycin (Figure S2E), which reduces TOR (target of rapamycin) signaling (Bjedov et al., 2010), or flies under dietary restriction (Chapman and Partridge, 1996; Figure S2F) and was even slightly downregulated in *mth*^{1} mutant flies (Lin et al., 1998; Figure S2G). Thus, circSfl expression was specifically increased in long-lived insulin mutant flies, and this increase was dependent on the transcription factor dFoxo, which is required for reduced insulin signaling to extend the lifespan.

Generation of circRNA Overexpression Mutants

Next, we aimed to generate circSfl overexpression mutants. We tested three different constructs (Figure 3A) to overexpress circSfl: circSfl-exon (only the exon that gives rise to circSfl), circSfl-1,000 (the circSfl exon plus 1,000 bp of the endogenous upstream and downstream introns), and circSfl-inverted (circSfl-inv; 1,000 bp of the endogenous upstream intron, which was copied, reverse complemented, and inserted as an artificial downstream intron), similar to a previous approach to artificially express human CDR1as in zebrafish (Memczak et al., 2013). Strikingly, circSfl-inv, but not circSfl exon or circSfl-1,000, was able to induce strong overexpression of circSfl by around 120-fold using the ubiquitous *da-Gal4* driver (circSfl-inv mutants are hereafter referred to as circSfl overexpression mutants) (Figure 3B). The linear transcripts Sfl RA and RB were largely unaffected; we only noticed a small upregulation of Sfl RB in circSfl overexpression mutants, but this was not reproducible in other qRT-PCR experiments. To verify that our overexpression system is universally applicable to other circRNAs, we generated similar overexpression constructs for circBtsz. In line with our results for circSfl, circBtsz-inv mutants also strongly overexpressed circBtsz, in contrast to circBtsz-exon or circBtsz-1,000, which did not affect circBtsz expression levels (Figures S3A and S3B). Altogether, we strongly overexpressed specific circRNAs *in vivo* in *Drosophila* by using engineered reverse-complementary flanking introns.

Overexpression of circSfl Extends the Lifespan

Because circSfl was consistently upregulated in diverse long-lived insulin mutant flies, we wondered whether overexpression of circSfl alone is sufficient to extend the lifespan. circSfl was upregulated in all four tested tissues under low-insulin conditions but showed the strongest upregulation in muscle and brain tissue. Therefore, we chose the neuron-specific *elav-Gal4* and the muscle-specific *MHC-Gal4* for tissue-specific overexpression and the *da-Gal4* driver line for ubiquitous overexpression. Strikingly, overexpression of circSfl with all three Gal4 driver lines resulted in significant lifespan extension in female flies (Figure 3C). The strongest lifespan extension was observed with ubiquitous overexpression of circSfl, where the median lifespan was increased by 15%. A mild but significant lifespan extension (4.5%) was induced by overexpression of circSfl in neurons. Muscle-specific overexpression of circSfl increased the median lifespan by 12%. In contrast, gut-specific overexpression of circSfl by NP1-Gal4 in females or ubiquitous overexpression of circSfl in males did not significantly change the lifespan of flies (Figures S4A and S4B), suggesting tissue- and sex-specific

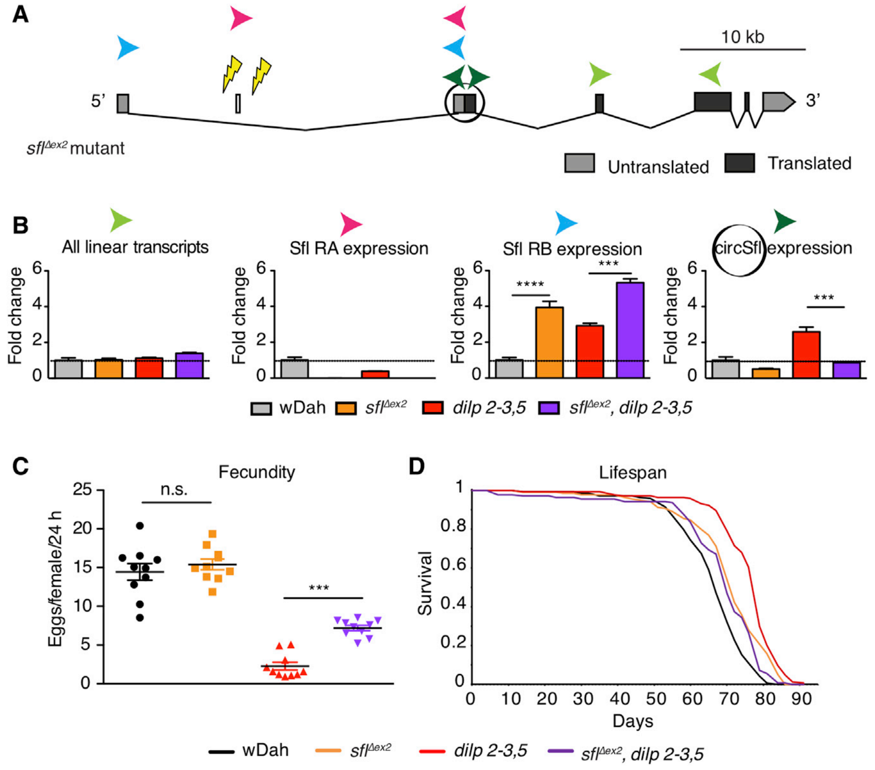

Figure 4. Backsplicing of the *sfl* circRNA Depends on the Presence of an Upstream Non-coding Exon, which Is Essential for IIS-Mediated Lifespan Extension

(A) Scheme of the mutated $sfl^{\Delta ex2}$ gene locus. The sequence deleted by CRISPR-Cas9 in $sfl^{\Delta ex2}$ mutant flies is indicated by yellow flashes. The primers used for qRT-PCR in (B) of the different *sfl* transcripts are indicated by arrowheads. The exon forming circSfl is indicated by a circle.

(B) Total linear Sfl RNA levels were not affected by removal of *sfl* exon 2. As expected, Sfl RA transcripts were absent in flies containing the $sfl^{\Delta ex2}$ deletion, and Sfl RB transcript levels were significantly upregulated in these flies. Importantly, circSfl expression was decreased in $sfl^{\Delta ex2}$ mutant flies, and the increased expression of circSfl in *dilp 2-3,5* mutants was reversed to wild-type levels in $sfl^{\Delta ex2}$*, dilp 2-3,5* double-mutant flies (***$p < 0.001$, ****$p < 0.0001$, 2-way-ANOVA with Bonferroni post hoc test, n = 3, mean ± SEM).

(C and D) A block in circSfl upregulation upon deletion of *sfl* exon 2 rescued the reduced fecundity (C) and increased lifespan (D) of insulin mutant flies.

(C) $sfl^{\Delta ex2}$ mutants showed wild-type fecundity. The low fecundity of *dilp 2-3,5* mutants was partially rescued in $sfl^{\Delta ex2}$*, dilp2-3,5* double mutants (***$p < 0.001$; interaction, **$p < 0.01$; 2-way-ANOVA, Bonferroni post hoc test, n = 10, mean ± SEM).

(D) Lifespan extension of *dilp 2-3,5* mutants was reduced in flies carrying the $sfl^{\Delta ex2}$ allele (wDah versus *dilp 2-3,5*: $p < 0.0001$, $sfl^{\Delta ex2}$ versus *dilp 2-3,5*: $p < 0.0001$ log rank test; interaction between $sfl^{\Delta ex2}$ and *dilp2-3,5*: $p < 0.0001$, Cox proportional hazard analysis). The $sfl^{\Delta ex2}$ lifespan of $sfl^{\Delta ex2}$ single mutants was not reduced.

See also Figure S5.

effects of circSfl on lifespan. Noteworthy, although there was leaky expression of circSfl in upstream activating sequence (UAS) control flies (*UAS-circSfl/+*), as measured by qRT-PCR (Figure 3B), these flies were not long lived compared with the Gal4 control, suggesting that, in a wild-type background, strong overexpression of circSfl is required for lifespan extension. Interestingly, although circSfl-overexpressing flies were long lived, unlike insulin mutant flies, they did not show increased stress resistance or climbing ability, a smaller size, or delayed development (Figures S4C–S4G), suggesting that circSfl affects a mechanism that specifically affects the lifespan but not the other, pleiotropic consequences of reduced insulin signaling. In summary, we demonstrated that circSfl is functional *in vivo* and plays an important role in the aging process. Our study shows that circRNAs can extend the lifespan and will contribute to the understanding of circRNA function *in vivo*.

Insulin-Mediated Lifespan Extension Is Dependent on Sfl RA and circSfl

Because circSfl overexpression was sufficient to extend the lifespan of fruit flies, we wondered whether circSfl is also required for lifespan extension of insulin mutant flies. Hence, we tried to knock down circSfl with small interfering RNAs (siRNAs) that target the circSfl-specific splice junction (Figure S5A), a strategy that has been used previously *in vitro* specifically to knock down circRNAs without affecting linear transcripts (Jeck et al., 2013). However, none of the three tested siRNAs was able to significantly reduce circSfl expression or to affect the linear transcripts RA and RB (Figure S5B). Although we did not identify a siRNA that downregulates circSfl, we were able to specifically knock down another circRNA we identified in our screen (circBtsz) with siRNAs (Figure S5C), indicating that siRNAs are, in principle, suitable for targeting circRNA expression *in vivo*. Because only the backsplice junction is unique to circRNA, siRNA design is very limited to this small area and might not always lead to successful knockdown. Next, we aimed to alter circSfl expression by affecting the alternative splicing of the host gene. circRNAs can compete or interact with splicing of the linear transcripts of their host genes (Ashwal-Fluss et al., 2014), and we found that circSfl backsplicing and alternative splicing of the linear Sfl RB transcript (but not RA) were correlated in the long-lived *dilp 2-3,5* mutants (Figure 2B). To address the link between circSfl backsplicing and alternative splicing of the linear Sfl transcripts and to test whether we can modulate the circRNA expression level by affecting alternative splicing, we generated $sfl^{\Delta ex2}$ mutant flies by CRISPR-Cas9. $sfl^{\Delta ex2}$ mutant flies lack ~250 bp of the 5′ UTR, including the 37-bp-long exon 2, which is unique to Sfl RA (Figure 4A); therefore, $sfl^{\Delta ex2}$ mutants express only the Sfl RB isoform. qRT-PCR demonstrated that total linear Sfl transcript levels were not affected in $sfl^{\Delta ex2}$ or *dilp 2-3,5* mutants and, as expected, that the Sfl RA isoform was not detectable in $sfl^{\Delta ex2}$ mutants (Figure 4B). Moreover, we found a 4-fold upregulation of the Sfl RB isoform in $sfl^{\Delta ex2}$

CellPress
OPEN ACCESS

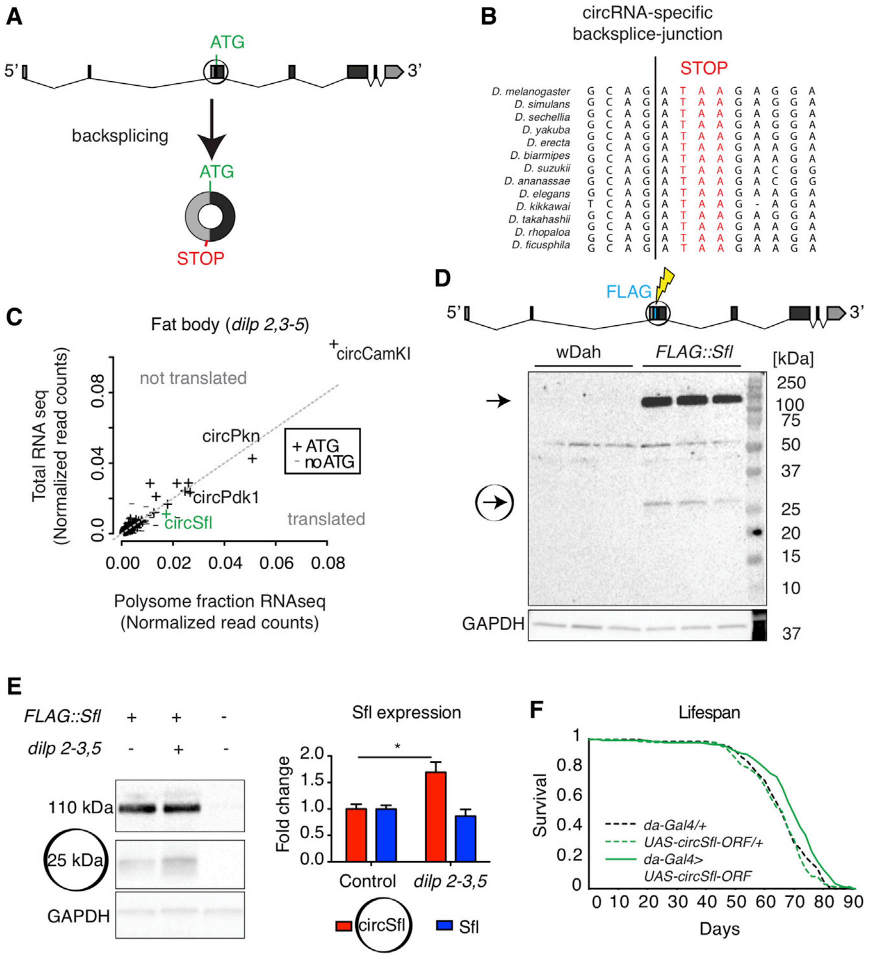

Figure 5. CircSfl Is Translated into a Protein that Is Upregulated in Insulin Mutant Flies

(A) circSfl shares the ATG start codon with its linear RNA isoforms and encodes an in-frame stop codon directly after the backsplice junction.

(B) Evolutionary conservation of the circSfl-specific stop codon among *Drosophila* species.

(C) Polysome profiling of *dilp2-3,5* mutant flies identified circSfl as a potentially translated circRNA. (+) indicates circRNAs that include the ATG start codon of their host genes.

(D) CRISPR-mediated knockin of a FLAG tag to the N terminus of Sfl and circSfl (*FLAG::Sfl* mutants) resulted in two specific bands in a western blot analysis that were absent in *wDah* wild-type flies that do not carry a FLAG tag (heads, n = 3). The 110-kDa band corresponds to the Sfl full-length protein, and the 25-kDa protein corresponds to the protein size encoded by circSfl.

(E) The 25-kDa protein, but not the 110 kDa Sfl protein variant, was increased in insulin mutant flies (*$p < 0.05$, Student's t test, n = 4, mean ± SEM).

(F) Ubiquitous overexpression of the circSfl open reading frame (ORF) from a linear transcript extends the lifespan of female flies (overexpressor versus *da*-Gal4 driver or UAS control, *****$p < 0.0001$; log-rank test, n = ~150).

See also Figure S6 and Data S3.

mutants, potentially compensating for the loss of Sfl RA. Interestingly, *sfl*$^{\Delta ex2}$, *dilp 2-3,5* double-mutant flies showed even higher levels of the Sfl RB isoform compared with the single-mutant flies, indicating that upregulation of Sfl RB in *sfl*$^{\Delta ex2}$ and *dilp 2-3,5* mutant flies is regulated by independent mechanisms (Figure 4B). Finally, circSfl expression was reduced by 50% in *sfl*$^{\Delta ex2}$ mutant flies, and the upregulation of circSfl in *dilp 2-3,5* mutants was abolished in *sfl*$^{\Delta ex2}$, *dilp 2-3,5* double mutants (Figure 4B). Our results suggest that upregulation of circSfl in *dilp 2-3,5* mutants depends on Sfl RA, verifying that there is indeed a link between backsplicing of circSfl and alternative splicing of linear *sfl* transcripts. Furthermore, *sfl*$^{\Delta ex2}$mutants may be used as circSfl knockdown flies because circSfl expression is reduced in these mutants. Therefore, we characterized *sfl*$^{\Delta ex2}$ as well as *sfl*$^{\Delta ex2}$, *dilp 2-3,5* double-mutant flies to test for genetic epistasis between *sfl* and *dilp 2-3,5*. Interestingly, the *dilp 2-3,5*-mediated reduction in egg laying was partially rescued in *sfl*$^{\Delta ex2}$, *dilp 2-3,5* double-mutant flies (2-way ANOVA interaction, $p < 0.01$), although *sfl*$^{\Delta ex2}$ single mutants showed normal egg-laying behavior (Figure 4C). Similarly, the smaller size of *dilp 2-3,5* mutant flies was partially rescued in *sfl*$^{\Delta ex2}$, *dilp 2-3,5* double mutants (Figure S5D), suggesting that the fecundity and size phenotypes of *dilp 2-3,5* mutants are, in part, dependent on sfl RA and circSfl. In contrast, *sfl*$^{\Delta ex2}$ mutants were developmentally delayed, and the development of *sfl*$^{\Delta ex2}$, *dilp 2-3,5* mutant flies was even more prolonged compared with *dilp 2-3,5* mutants (Figure S5E), pointing toward independent underlying mechanisms. Finally, the lifespan extension of *dilp 2-3,5* mutants was strongly reduced in *sfl*$^{\Delta ex2}$, *dilp 2-3,5* double mutants ($p < 0.0001$, Cox proportional hazard analysis), although *sfl*$^{\Delta ex2}$ single mutants were not short lived but, rather, slightly long lived (Figure 4D). We demonstrated that the reduced size, reduced fecundity, and extended lifespan, but not the delayed development of *dilp 2-3,5* mutants, are dependent on Sfl RA and circSfl.

CircSfl Is Translated and Dynamically Regulated

We have demonstrated that circSfl is functional *in vivo* because overexpression of circSfl extends the lifespan and that circSfl biogenesis is linked to alternative splicing of its host gene. However, the molecular function of circSfl is still unclear. Some circRNAs have been suggested to sponge microRNAs (miRNAs); e.g., the human CDR1as circRNA contains around 70 binding sites for miR-7 (Hansen et al., 2013; Memczak et al., 2013). In contrast, we only identified 10 miRNA seed sequences of 9 different miRNAs in the sequence of circSfl (Figure S6A), which is a relatively low number compared with other *Drosophila* circRNAs (Figure S6B). This lack of enrichment of miRNA binding sites makes it unlikely that circSfl acts as a miRNA sponge. It has been shown recently that some circRNAs can be translated into proteins when they harbor the ATG start codon of their host genes and an in-frame stop codon after the backsplice junction (Legnini et al., 2017; Pamudurti et al., 2017). Interestingly, the ATG start codon of the canonical Sfl ORF is part of circSfl and could therefore be

used for translation of circSfl (Figure 5A). In addition, the first codon in frame after the circRNA-specific splice junction is a stop codon. Remarkably, the stop codon after the circRNA-specific splice junction is conserved in many *Drosophila* species (Figure 5B). The evolutionary conservation of this stop codon for several million years suggests that it might be functional. If circSfl was indeed translated, then it would be identical to the N terminus of the Sfl full-length protein encoded by the canonical, linear transcripts, including the complete transmembrane domain, but lack the enzymatically active domain at the C terminus of the Sfl full-length protein. To determine whether the circSfl RNA transcript is associated with the translation machinery, we analyzed its presence in polysome profiles compared with total RNA sequencing (RNA-seq) of wild-type and *dilp 2-3,5* fat bodies (Data S3). Similar to Pamudurti et al. (2017), we observed that circRNAs that include the ATG start codon of the host gene are among the most highly expressed circRNAs in polysome-enriched RNA samples; e.g., circCamKI, circPkn, and circPdk1 (Figures 5C and S6C). Furthermore, we detected circSfl transcripts in fat bodies of wild-type, polysome-enriched RNA samples (Figure S6C) and as one of the most highly detected circRNAs in polysome-enriched RNA samples in *dilp 2-3,5* fat bodies (Figure 5C), further suggesting that circSfl might be translated.

To test whether circSfl is indeed translated *in vivo*, we used CRISPR-Cas9 to generate *FLAG::Sfl* mutant flies. We inserted a FLAG tag into the endogenous *sfl* gene, tagging the full-length Sfl protein of the host gene at the N terminus and also the protein that might arise from circSfl (Figure 5D). Indeed, when analyzing *FLAG::Sfl* mutants by western blotting, we observed two distinct bands corresponding to a molecular weight of around 110 kDa (Sfl full-length) and 25 kDa (Figure 5D). Based on its sequence, translation of the circSfl ORF, including the FLAG tag, should result in a 24.75-kDa protein. However, this band might also arise from an unknown shorter isoform of Sfl or a Sfl degradation product. Therefore, we modified our previously generated circSfl-overexpressing flies and generated another mutant that overexpressed and FLAG-tagged only the circSfl protein but not the full-length Sfl protein (referred to as *FLAG::circSfl*). Western blotting demonstrated that, indeed, overexpression of *FLAG::circSfl* led to a detectable protein with a size of around 25 kDa (Figure S6D). However, we noticed that this protein band ran slightly faster on SDS-PAGE compared with the *FLAG::Sfl* knockin mutants, suggesting a smaller size of this protein or different post-translational modifications from the protein band originating from *FLAG::Sfl* knockin flies in comparison with the overexpressed protein. To further verify that the smaller band indeed corresponded to the translated circRNA and not to a degradation product of the canonical protein, we used *UAS-sfl* flies, as published previously (Yamamoto-Hino et al., 2012), which have an N-terminal hemagglutinin (HA) tag and express the cDNA of Sfl without flanking introns, generating only linear Sfl transcripts, but not circSfl (Figure S6E). Western blot analysis showed only one protein band, corresponding to the full-length protein, and no smaller band at around 25 kDa, even at high exposure, in *elav-Gal4*>*UAS-sfl* flies (Figure S6F). It is tempting to speculate that the circSfl protein might play a role in insulin-mediated lifespan extension because circSfl RNA was upregulated in insulin mutant flies based on qRT-PCR. Therefore, we tested whether the circSfl protein was also upregulated in *dilp 2-3,5* mutant flies, and, indeed, circSfl protein levels increased significantly in *dilp 2-3,5* mutants (Figure 5E). Finally, to test whether the protein, which is encoded by circSfl, is sufficient to extend the lifespan, we generated a transgenic fly line that overexpresses the circSfl ORF from a linear transcript. Remarkably, ubiquitous overexpression of the linear circSfl ORF significantly extend the lifespan of female flies. Thus, multiple lines of evidence indicate that circSfl is translated and that the encoded protein is sufficient for lifespan extension.

Neuron-Specific Overexpression of Sfl Extends the Lifespan

If circSfl is indeed translated into a small protein, then it would be identical to the N terminus of the Sfl full-length protein originating from the canonical, linear transcripts. The *sfl* linear transcripts encode for an N-deacetylase/N-sulfotransferase (Ndst) that catalyzes synthesis of heparan sulfate (a glycosaminoglycan) by sulfation of the N and 6-O position of N-acetylglucosamine (GlcNAc) (Toyoda et al., 2000; Lin and Perrimon, 1999) in the Golgi apparatus (Yano et al., 2005). Interestingly, the circSfl ORF covers the full cytoplasmic N terminus of Sfl as well as the hydrophobic transmembrane domain but lacks the luminal enzymatic domain important for synthesis of heparan sulfate. Because overexpression of the protein encoded by circSfl extended the lifespan, we next tested whether overexpression of the Sfl full-length protein might also affect the lifespan. Using previously published *UAS-sfl* transgenic flies (Yamamoto-Hino et al., 2012) to overexpress Sfl in the muscle, gut, neurons, or ubiquitously, we found that overexpression of Sfl in the muscle or gut did not affect the lifespan and that ubiquitous overexpression was detrimental for the lifespan (Figures 6A, 6B, and S4A). However, neuron-specific overexpression of Sfl by *elav-Gal4* led to a lifespan extension of about 15% in females flies (Figure 6C). Consistent with the results obtained with circSfl, overexpression of Sfl did not increase the lifespan in male flies (Figure S4B). Surprisingly, neuron-specific overexpression of Sfl in the *dilp 2-3,5* mutant background decreased the lifespan (Figure 6D), suggesting that Sfl and *dilp 2-3,5* interact genetically with each other. However, similar to circSfl overexpression mutants, we did not observe any differences in starvation stress resistance, climbing ability, weight, or development in Sfl-overexpressing flies (Figures S7C–S7F). In contrast, we observed that ubiquitous knockdown of Sfl by *da-Gal4* is developmentally lethal in late pupal stages (data not shown), whereas adult-onset, ubiquitous knockdown of Sfl by *da-GeneSwitch* (*da-GS*) shortened the lifespan (Figure S7G). In summary, circSfl and Sfl can significantly extend the lifespan, but we observed tissue-specific differences. The strongest lifespan extension by circSfl was observed with ubiquitous overexpression, but the only, and strong, lifespan extension by Sfl was observed with neuron-specific overexpression. These results suggest that circSfl and Sfl might share some functions but also have independent roles, which is in line with the observation that circSfl protein is identical to the cytoplasmic and transmembrane domain of Sfl but lacks the C terminus in the Golgi.

CellPress
OPEN ACCESS

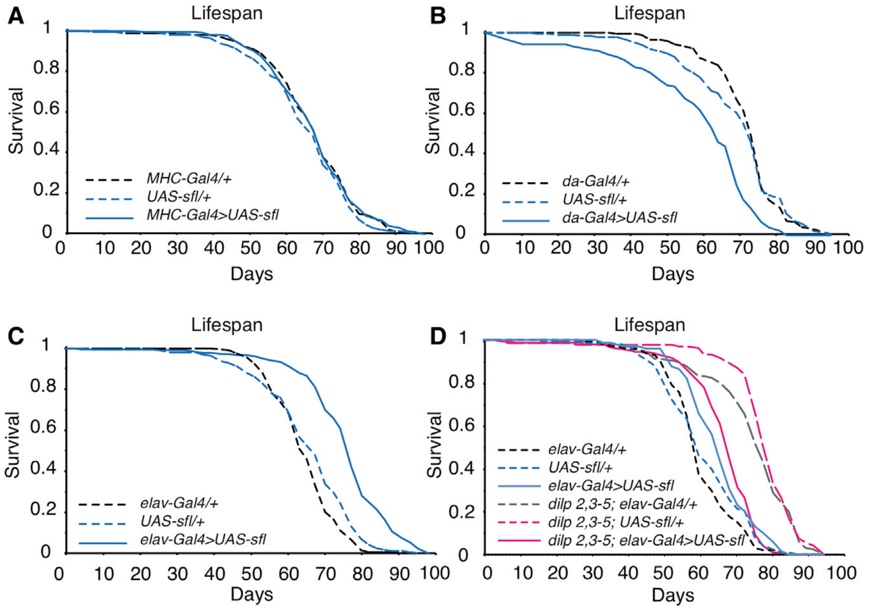

Figure 6. Neuron-Specific Overexpression of Sfl Extends the Lifespan of *Drosophila*

(A) Muscle-specific overexpression of Sfl did not affect the lifespan.

(B) Ubiquitous overexpression of Sfl shortened the lifespan (overexpressor versus driver or UAS control, $p < 0.0001$; log rank test, n = ~200).

(C) Overexpression of Sfl in neurons by *elav-Gal4* extended the lifespan (overexpressor versus driver or UAS control, $p < 0.0001$; log rank test, n = ~200).

(D) Neuron-specific overexpression of Sfl in the *dilp 2-3,5* mutant background shortened the *dilp 2-3,5*-mediated lifespan extension (interaction of Sfl overexpression with *dilp 2-3,5*, $p < 0.0001$; Cox proportional hazard analysis, n = ~80–150).

Female flies were used. See also Figure S7.

Does Dally Mediate Lifespan Extension Downstream of Sfl?

Sfl mediates synthesis of heparan sulfate, and in flies there are four heparan sulfate proteoglycans that are targeted by Sfl: Dally, Dally-like (Dlp), Syndecan (Sdc), and Perlecan (Lin and Perrimon, 1999; Baeg et al., 2004). To test whether heparan sulfate proteoglycans mediated the effect of Sfl on lifespan, we overexpressed Dally, Dlp, and Sdc in neurons using commercially available UAS lines. Strikingly, Dally but not Sdc or Dlp overexpression, extended the lifespan to a similar extent as Sfl (Figures 7A–7C), suggesting that Dally may be the target through which Sfl overexpression extends the lifespan. To verify this further, we conducted genetic epistasis experiments and used RNAi to knock down Dally in Sfl-overexpressing flies. Strikingly, Dally knockdown abolished the Sfl-induced lifespan extension (Figure 7D), suggesting that Dally is a crucial downstream target of Sfl that mediates the lifespan extension. In summary, we showed that circSfl and Sfl linear splicing is altered in insulin mutant flies and that circSfl and Sfl overexpression can extend the lifespan in a tissue-specific manner.

DISCUSSION

One of the most striking discoveries about circRNAs is the observation that they accumulate with age in neuronal tissues of diverse species (Cortés-López et al., 2018; Gruner et al., 2016; Westholm et al., 2014; Hall et al., 2017). Several hypotheses regarding why circRNAs accumulate with age have been proposed. First, circRNAs are more stable compared with linear RNA molecules (Cocquerelle et al., 1993). Second, it has been suggested that circRNAs accumulate with age specifically in neuronal tissue because neurons are mostly post-mitotic, and, therefore, the more stable circRNAs are not degraded by proliferation or cell death (Knupp and Miura, 2018). However, if this theory holds true, then circRNAs should accumulate in most tissues of the fruit fly because *Drosophila* is a mainly post-mitotic organism. Third, alternative splicing is increased and more error prone with age (Mazin et al., 2013; Rodríguez et al., 2016), potentially resulting in more backsplicing of circRNAs. In our study, we showed that circRNA accumulation with age is slowed down in long-lived insulin mutants. This might point toward the third theory of why circRNAs accumulate with age and is supported by the finding that the splicing factor SFA-1 is required for dietary restriction-induced longevity in nematodes (Heintz et al., 2017), highlighting the importance of splicing for lifespan extension upon deregulated nutrient sensing. Our findings demonstrated that accumulation of circRNAs with age is malleable, suggesting that accumulation of circRNAs might be a potential aging biomarker.

In our study, we identified several circRNAs that were differentially regulated in response to reduced insulin signaling in *dilp 2-3,5* mutants, including circSfl. circSfl was also upregulated in two other insulin mutant flies that have an extended lifespan, and the upregulation is dependent on the dFoxo transcription factor, an essential mediator of longevity downstream of IIS (Grönke et al., 2010; Demontis and Perrimon, 2010; Broughton et al., 2005; Slack et al., 2011). Notably, the magnitude of upregulation of circSfl in these mutants was correlated with the magnitude of the lifespan extension, with strong, up to 7-fold upregulation in the very long-lived *dilp 2-3,5* mutants and only mildly upregulated in MNC-ablated flies and dFoxo overexpression flies (1.5- to 2-fold), which show a more mild lifespan extension (Grönke et al., 2010; Demontis and Perrimon, 2010; Broughton et al., 2005; Slack et al., 2011). Similarly, the linear transcript Sfl RB was only upregulated in *dilp2-3,5* mutant flies and not in the two other insulin mutants, suggesting that longevity and expression of the linear isoform can be uncoupled. Interestingly, neither circSfl nor the linear Sfl isoforms are upregulated upon rapamycin treatment (Bjedov et al., 2010) or dietary restriction (Chapman and Partridge, 1996) or in *mth*^{1} mutant flies (Lin et al., 1998), suggesting that upregulation of circSfl is not a general hallmark of lifespan-extending interventions in flies but specific to IIS-mediated longevity.

To overexpress circRNAs *in vivo*, we tested different UAS constructs based on previous publications (Ashwal-Fluss et al., 2014; Memczak et al., 2013; Pamudurti et al., 2017). As expected, overexpression of the circRNA exon without its flanking introns did not lead to increased circRNA expression because

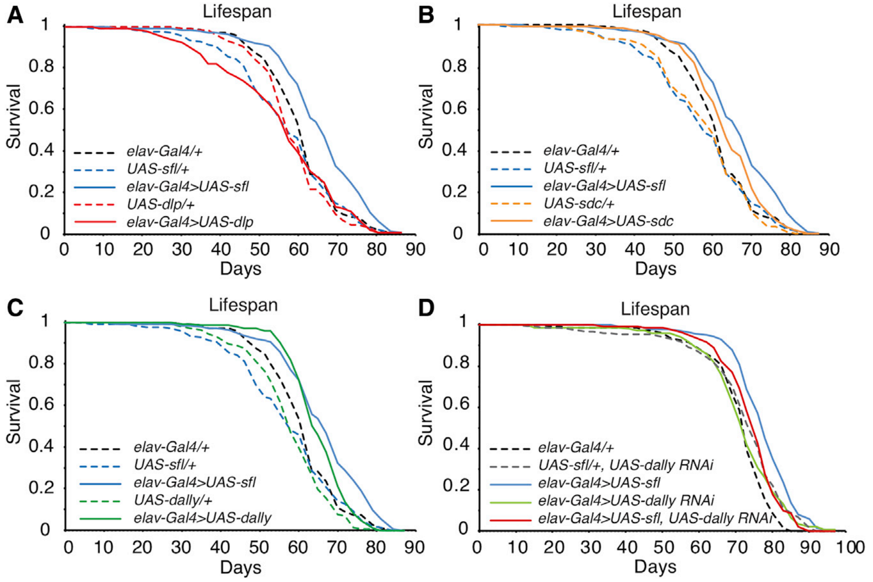

Figure 7. The Heparan Sulfate Proteoglycan Dally Mediates Sfl-Induced Lifespan Extension

(A and B) Overexpression of the heparan sulfate proteoglycans (A) Dally-like protein (Dlp) and (B) Syndecan (Sdc) did not extend the lifespan compared with the *elav-Gal4/+* driver control.

(C) Overexpression of Dally extended the lifespan (Dally overexpressor versus Gal4 control, $p < 0.05$; Dally overexpressor versus UAS control, $p < 0.01$; log rank test, n = ~150) to a similar extent as overexpression of Sfl.

(D) Lifespan extension mediated by Sfl overexpression was partially rescued by Dally RNAi (*elav-Gal4>UAS-sfl* versus *elav-Gal4>UAS-sfl, dally RNAi*: $p < 0.001$; *elav-Gal4/+* versus *elav-Gal4>UAS-sfl, dally RNAi*: $p < 0.001$, log rank test; interaction between Sfl overexpression and Dally RNAi: $p < 0.0001$, Cox proportional hazard analysis; n = ~150).

The lifespans shown in (A)–(C) were determined in parallel, and the same *elav-Gal4/+*, *UAS-sfl/+* and *elav-Gal4>UAS-sfl* control lifespans are shown in (A)–(C) to allow direct comparison.

flanking introns are required for biogenesis of circRNAs (Kramer et al., 2015; Ashwal-Fluss et al., 2014). In contrast, introducing reverse complementary matching flanking introns strongly increased biogenesis of circRNAs *in vivo*. Our results are in line with previous studies that expressed circRNAs by engineering reverse complementary introns in zebrafish (Memczak et al., 2013) and *in vitro* (Liang and Wilusz, 2014; Tatomer et al., 2017; Kramer et al., 2015; Liu et al., 2018). However, the first study that overexpressed a circRNA (circMbl) *in vivo* in *Drosophila* used a minigene construct including the circMbl exon and around 100 bp of the natural flanking introns but no inverted repeats. CircMbl overexpression led to a 4-fold increase in the circMbl expression level (Pamudurti et al., 2017), much less than the strong overexpression we achieved by engineered flanking introns. In summary, our mutants demonstrate that circRNAs can be efficiently overexpressed in *Drosophila* using engineered, reverse complementary matches in flanking introns that increase circRNA biogenesis *in vivo*.

Furthermore, we generated $sfl^{\Delta ex2}$ mutant flies that lack the Sfl RA-specific exon 2 and demonstrated that circSfl biogenesis is dependent on Sfl RA. Combination of $sfl^{\Delta ex2}$ mutants with *dilp 2-3,5* mutants revealed that the lifespan extension of *dilp 2-3,5* mutants is partly dependent on the presence of this exon. The biogenesis of circRNAs is poorly understood, but several RNA binding proteins have been shown recently to inhibit or promote circularization, including Muscleblind, Quaking, and Adar1 (Conn et al., 2015; Ivanov et al., 2015; Rybak-Wolf et al., 2015; Ashwal-Fluss et al., 2014). It is tempting to speculate that an RNA binding protein might bind to the Sfl RA-specific exon and promote biogenesis of circSfl, which is abolished in $sfl^{\Delta ex2}$ mutant flies. Because $sfl^{\Delta ex2}$ mutants affect biogenesis of circSfl and expression of the linear Sfl RA isoform, we can currently not formally exclude a role of the linear splice variant in insulin-mediated longevity. However, several lines of evidence suggest circSfl as the causal factor in this context. First, although Sfl RA expression was lost in $sfl^{\Delta ex2}$ mutants, overall expression of linear Sfl was not affected. This is consistent with the finding that Sfl protein levels were not changed in *dilp 2-3,5* mutant flies despite differential alternative splicing of the RA and RB isoforms. Thus, modifying exon 2 expression levels does not seem to affect Sfl protein levels, which can affect the lifespan. In addition, overexpression of circSfl and a linear transcript encoding the circSfl protein was sufficient to extend the lifespan, directly linking circSfl expression with longevity regulation. Given that most circRNAs are embedded in a host gene, generation of specific circRNA mutants that do not affect the host gene has been very challenging in the field. Because siRNA-mediated knockdown was not efficient in the case of circSfl, in new strategies should be tested in the future (e.g., by modification or deletion of the flanking introns that affect circRNA biogenesis), which can then be used to verify our hypothesis.

We presented several lines of evidence showing that circSfl might be translated into a protein that is identical to the N terminus arising from linear Sfl transcripts. Sequence homology analysis showed that the in-frame stop codon after the circRNA-specific backsplice junction is conserved within *Drosophila* species that are separated by 10–20 million years of evolution, suggesting that the protein encoded by circSfl might also be conserved between these species. However, we were not able to detect the identical stop codon in more distantly related insect species, like honeybees or mosquitoes (data not shown), which could indicate that the circlSfl protein is specific to *Drosophila* species or that other stop codons more downstream are used in other insects. Pamudurti et al. (2017) previously identified 37 potentially translated circRNAs in *Drosophila* using ribosome footprinting on wild-type *Drosophila* heads; however, they failed to detect circSfl. Similarly, in our polysome profiling experiment, only very few circSfl reads were detected in wild-type fat bodies. In contrast, in *dilp 2-3,5* mutant fat bodies, circSfl was one of the most abundant circRNAs, consistent with the insulin-dependent increase in circSfl transcript and protein levels. Furthermore, we have shown that the protein encoded by circSfl and the protein

arising from the linear Sfl transcripts can positively affect the lifespan of flies. This finding might indicate that both proteins affect the lifespan through overlapping mechanisms or by interacting with each other. Because the protein encoded by circSfl lacks the catalytic domain, it is unlikely that it acts as an active enzyme. Thus, circSfl may interact with proteins similar to the Sfl full-length protein in the cytoplasm or the membrane. For example, one could imagine that circSfl might interact with a repressor of the Sfl full-length protein, promoting the activity of Sfl and extending the lifespan. Alternatively, the truncated circSfl protein could also act as a dominant-negative protein because Sfl overexpression has also been suggested to cause a loss-of-function phenotype (Kamimura, Maeda and Nakato, 2011). Noteworthy is that overexpression of circSfl and Sfl caused tissue-specific effects on longevity, which could indicate that they work via different mechanisms or that different expression levels in different tissues are needed for the beneficial effects of the two proteins on lifespan. Interestingly, overexpression of circSfl and Sfl only extended the female but not the male lifespan despite upregulation of circSfl in dilp2-3,5 mutant males. This might reflect the gender bias in insulin-mediated longevity, in which females often show stronger effects than males (Austad and Fischer, 2016).

The *sfl* gene in *Drosophila* encodes for an Ndst and catalyzes synthesis of heparan sulfate (a glycosaminoglycan) by sulfation of the N and 6-O position of GlcNAc (Toyoda et al., 2000). Heparan sulfate is essential for wingless and fibroblast growth factor (FGF) receptor signaling, and full knockout of *sfl* is embryonic lethal (Lin and Perrimon, 1999). Sfl has been suggested to be localized to the Golgi apparatus (Yano et al., 2005; Yamamoto-Hino et al., 2012) and may be involved in the unfolded protein response (He et al., 2014). Furthermore, knockdown of Sfl increased the autophagy machinery and ubiquitinated proteins (Reynolds-Peterson et al., 2017) and reduced the climbing ability of flies (Webber et al., 2018), suggesting that Sfl is required for protein homeostasis and health. Similarly, we demonstrated that neuron-specific knockdown of Sfl is detrimental for the lifespan but that neuron-specific overexpression of Sfl extends the lifespan. Furthermore, we demonstrated, by genetic epistasis experiments, that Dally might contribute to the Sfl-mediated lifespan extension. Previous studies have demonstrated that overexpression of Sfl increases heparan sulfate levels and disrupts normal Wingless (Wg) and Decapentaplegic (Dpp) signaling (Kamimura, Maeda and Nakato, 2011), with the latter being controlled by Dally (Fujise et al., 2003). However, similar to Sfl, the role of Dally has been characterized extensively during development (Ferreira and Milán, 2015; Zhang et al., 2013; Lin and Perrimon, 1999) but has not yet been implicated in aging.

In summary, we demonstrated that neuronal circRNA accumulation with age is malleable and reduced in long-lived insulin mutants. Furthermore, we established an efficient method to overexpress circRNAs *in vivo* by using reverse complementary introns. Interestingly, we showed that a single circRNA (circSfl) can extend the lifespan in *Drosophila*. We propose that circSfl is translated into a protein that shares the same N terminus with the full-length protein arising from linear transcripts and potentially similar functions. Our study will help to further elucidate the molecular mechanisms underlying longevity and provides unique insights into the *in vivo* function of circRNAs.

Limitations of the Study

A limitation of our study is that it is currently unclear whether the circRNA-derived peptide and the full-length Sfl protein affect the lifespan by the same or by independent mechanisms. Lifespan extension by the full-length Sfl protein is dependent on its direct downstream target, the Dally protein. Thus, to address whether lifespan extension upon circSfl overexpression works in a similar way and also requires Dally, we performed epistasis experiments by co-overexpression of the circSfl protein and dally RNAi and measured the lifespan of these flies. These experiments had to be terminated because of the current coronavirus crisis. In the future, it will be very interesting to further elucidate the mechanism of lifespan extension by circSfl and Sfl using genetic epistasis experiments.

STAR★METHODS

Detailed methods are provided in the online version of this paper and include the following:

- KEY RESOURCES TABLE
- RESOURCE AVAILABILITY
  - Lead Contact
  - Materials Availability
  - Data and Code Availability
- EXPERIMENTAL MODEL AND SUBJECT DETAILS
  - Fly husbandry
- METHOD DETAILS
  - Transgenic flies
  - Lifespan analysis
  - Fecundity assay
  - Stress assays
  - Development assay
  - Climbing assay
  - Molecular Cloning
  - RNA extraction, cDNA synthesis and q-RT-PCR
  - RNA sequencing
  - circRNA sequence analysis
  - RNase R treatment
  - Polysome profiling
  - Western blotting
- QUANTIFICATION AND STATISTICAL ANALYSIS

SUPPLEMENTAL INFORMATION

Supplemental Information can be found online at https://doi.org/10.1016/j.molcel.2020.06.011.

ACKNOWLEDGMENTS

We gratefully acknowledge Franziska Metge and Jorge Boucas of the Bioinformatics core facility at the Max Planck Institute for Biology of Ageing for help with data analysis and the Max Planck Genome Center Cologne for generation of sequencing libraries and performing next-generation sequencing. We acknowledge Ramona Hoppe and Andrea Müller-Hartmann for excellent technical assistance. We are grateful to Satoshi Goto for supplying the UAS-sfl fly line. The Bloomington *Drosophila* Stock Center (NIH P40OD018537) and

Vienna Drosophila Resource Center (VDRC) are acknowledged for fly lines. BAC clones containing *Drosophila* gDNA obtained from P[acman] Resources were used in this study. We acknowledge funding from the Max Planck Society, the Mary Gates Research Scholarship (to R.S.) and Bundesministerium für Bildung und Forschung grant SyBACol 0315893A-B (to L.S.T. and L.P.). The research leading to these results has received funding from the European Research Council under the European Union Seventh Framework Program (FP7/2007-2013)/ERC grant agreement 268739 (to L.P.).

AUTHOR CONTRIBUTIONS

Conceptualization, C.M.W., S.G., and L.P.; Methodology, C.M.W., R.S., J.E., A.P., and L.S.T.; Formal Analysis, J.C. and C.D.; Investigation, C.M.W., R.S., and S.G.; Resources, L.S.T.; Writing, C.M.W., S.G., and L.P.; Visualization, C.M.W.; Supervision, S.G. and L.P.; Project Administration, C.M.W., S.G., and L.P.; Funding Acquisition, L.P.

DECLARATION OF INTERESTS

The authors declare no competing interests.

Received: May 3, 2019
Revised: April 21, 2020
Accepted: June 4, 2020
Published: June 26, 2020

REFERENCES

Ashwal-Fluss, R., Meyer, M., Pamudurti, N.R., Ivanov, A., Bartok, O., Hanan, M., Evantal, N., Memczak, S., Rajewsky, N., and Kadener, S. (2014). circRNA biogenesis competes with pre-mRNA splicing. Mol. Cell *56*, 55–66.

Austad, S.N., and Fischer, K.E. (2016). Sex Differences in Lifespan. Cell Metab. *23*, 1022–1033.

Baeg, G.H., Selva, E.M., Goodman, R.M., Dasgupta, R., and Perrimon, N. (2004). The Wingless morphogen gradient is established by the cooperative action of Frizzled and Heparan Sulfate Proteoglycan receptors. Dev. Biol. *276*, 89–100.

Bass, T.M., Grandison, R.C., Wong, R., Martinez, P., Partridge, L., and Piper, M.D. (2007). Optimization of dietary restriction protocols in Drosophila. J. Gerontol. A Biol. Sci. Med. Sci. *62*, 1071–1081.

Bischof, J., Maeda, R.K., Hediger, M., Karch, F., and Basler, K. (2007). An optimized transgenesis system for Drosophila using germ-line-specific phiC31 integrases. Proc. Natl. Acad. Sci. USA *104*, 3312–3317.

Bjedov, I., Toivonen, J.M., Kerr, F., Slack, C., Jacobson, J., Foley, A., and Partridge, L. (2010). Mechanisms of life span extension by rapamycin in the fruit fly Drosophila melanogaster. Cell Metab. *11*, 35–46.

Broughton, S.J., Piper, M.D., Ikeya, T., Bass, T.M., Jacobson, J., Driege, Y., Martinez, P., Hafen, E., Withers, D.J., Leevers, S.J., and Partridge, L. (2005). Longer lifespan, altered metabolism, and stress resistance in Drosophila from ablation of cells making insulin-like ligands. Proc. Natl. Acad. Sci. USA *102*, 3105–3110.

Capel, B., Swain, A., Nicolis, S., Hacker, A., Walter, M., Koopman, P., Goodfellow, P., and Lovell-Badge, R. (1993). Circular transcripts of the testis-determining gene Sry in adult mouse testis. Cell *73*, 1019–1030.

Chapman, T., and Partridge, L. (1996). Female fitness in Drosophila melanogaster: an interaction between the effect of nutrition and of encounter rate with males. Proc. Biol. Sci. *263*, 755–759.

Cheng, J., Metge, F., and Dieterich, C. (2016). Specific identification and quantification of circular RNAs from sequencing data. Bioinformatics *32*, 1094–1096.

Clancy, D.J., Gems, D., Harshman, L.G., Oldham, S., Stocker, H., Hafen, E., Leevers, S.J., and Partridge, L. (2001). Extension of life-span by loss of CHICO, a Drosophila insulin receptor substrate protein. Science *292*, 104–106.

Cocquerelle, C., Mascrez, B., Hétuin, D., and Bailleul, B. (1993). Mis-splicing yields circular RNA molecules. FASEB J. *7*, 155–160.

Conn, S.J., Pillman, K.A., Toubia, J., Conn, V.M., Salmanidis, M., Phillips, C.A., Roslan, S., Schreiber, A.W., Gregory, P.A., and Goodall, G.J. (2015). The RNA binding protein quaking regulates formation of circRNAs. Cell *160*, 1125–1134.

Cortés-López, M., Gruner, M.R., Cooper, D.A., Gruner, H.N., Voda, A.I., van der Linden, A.M., and Miura, P. (2018). Global accumulation of circRNAs during aging in Caenorhabditis elegans. BMC Genomics *19*, 8.

Demontis, F., and Perrimon, N. (2010). FOXO/4E-BP signaling in Drosophila muscles regulates organism-wide proteostasis during aging. Cell *143*, 813–825.

Dinkova, T.D., Keiper, B.D., Korneeva, N.L., Aamodt, E.J., and Rhoads, R.E. (2005). Translation of a small subset of Caenorhabditis elegans mRNAs is dependent on a specific eukaryotic translation initiation factor 4E isoform. Mol. Cell. Biol. *25*, 100–113.

Edgar, R., Domrachev, M., and Lash, A.E. (2002). Gene Expression Omnibus: NCBI gene expression and hybridization array data repository. Nucleic Acids Res. *30*, 207–210.

Ferreira, A., and Milán, M. (2015). Dally Proteoglycan Mediates the Autonomous and Nonautonomous Effects on Tissue Growth Caused by Activation of the PI3K and TOR Pathways. PLoS Biol. *13*, e1002239.

Fujise, M., Takeo, S., Kamimura, K., Matsuo, T., Aigaki, T., Izumi, S., and Nakato, H. (2003). Dally regulates Dpp morphogen gradient formation in the Drosophila wing. Development *130*, 1515–1522.

Grabowski, P.J., Zaug, A.J., and Cech, T.R. (1981). The intervening sequence of the ribosomal RNA precursor is converted to a circular RNA in isolated nuclei of Tetrahymena. Cell *23*, 467–476.

Grant, C.E., Bailey, T.L., and Noble, W.S. (2011). FIMO: scanning for occurrences of a given motif. Bioinformatics *27*, 1017–1018.

Greene, J.C., Whitworth, A.J., Kuo, I., Andrews, L.A., Feany, M.B., and Pallanck, L.J. (2003). Mitochondrial pathology and apoptotic muscle degeneration in Drosophila parkin mutants. Proc. Natl. Acad. Sci. USA *100*, 4078–4083.

Grönke, S., Clarke, D.F., Broughton, S., Andrews, T.D., and Partridge, L. (2010). Molecular evolution and functional characterization of Drosophila insulin-like peptides. PLoS Genet. *6*, e1000857.

Gruner, H., Cortés-López, M., Cooper, D.A., Bauer, M., and Miura, P. (2016). CircRNA accumulation in the aging mouse brain. Sci. Rep. *6*, 38907.

Hall, H., Medina, P., Cooper, D.A., Escobedo, S.E., Rounds, J., Brennan, K.J., Vincent, C., Miura, P., Doerge, R., and Weake, V.M. (2017). Transcriptome profiling of aging Drosophila photoreceptors reveals gene expression trends that correlate with visual senescence. BMC Genomics *18*, 894.

Hansen, T.B., Jensen, T.I., Clausen, B.H., Bramsen, J.B., Finsen, B., Damgaard, C.K., and Kjems, J. (2013). Natural RNA circles function as efficient microRNA sponges. Nature *495*, 384–388.

He, B.Z., Ludwig, M.Z., Dickerson, D.A., Barse, L., Arun, B., Vilhjálmsson, B.J., Jiang, P., Park, S.Y., Tamarina, N.A., Selleck, S.B., et al. (2014). Effect of genetic variation in a Drosophila model of diabetes-associated misfolded human proinsulin. Genetics *196*, 557–567.

Heintz, C., Doktor, T.K., Lanjuin, A., Escoubas, C., Zhang, Y., Weir, H.J., Dutta, S., Silva-García, C.G., Bruun, G.H., Morantte, I., et al. (2017). Splicing factor 1 modulates dietary restriction and TORC1 pathway longevity in C. elegans. Nature *541*, 102–106.

Ivanov, A., Memczak, S., Wyler, E., Torti, F., Porath, H.T., Orejuela, M.R., Piechotta, M., Levanon, E.Y., Landthaler, M., Dieterich, C., and Rajewsky, N. (2015). Analysis of intron sequences reveals hallmarks of circular RNA biogenesis in animals. Cell Rep. *10*, 170–177.

Jeck, W.R., Sorrentino, J.A., Wang, K., Slevin, M.K., Burd, C.E., Liu, J., Marzluff, W.F., and Sharpless, N.E. (2013). Circular RNAs are abundant, conserved, and associated with ALU repeats. RNA *19*, 141–157.

Kamimura, K., Maeda, N., and Nakato, H. (2011). In vivo manipulation of heparan sulfate structure and its effect on Drosophila development. Glycobiology *21*, 607–618.

Kent, W.J., Sugnet, C.W., Furey, T.S., Roskin, K.M., Pringle, T.H., Zahler, A.M., and Haussler, D. (2002). The human genome browser at UCSC. Genome Res. *12*, 996–1006.

CellPress
OPEN ACCESS

Kenyon, C., Chang, J., Gensch, E., Rudner, A., and Tabtiang, R. (1993). A C. elegans mutant that lives twice as long as wild type. Nature *366*, 461–464.

Kim, D., Langmead, B., and Salzberg, S.L. (2015). HISAT: a fast spliced aligner with low memory requirements. Nat. Methods *12*, 357–360.

Kleaveland, B., Shi, C.Y., Stefano, J., and Bartel, D.P. (2018). A Network of Noncoding Regulatory RNAs Acts in the Mammalian Brain. Cell *174*, 350–362.e17.

Knupp, D., and Miura, P. (2018). CircRNA accumulation: A new hallmark of aging? Mech. Ageing Dev. *173*, 71–79.

Kramer, M.C., Liang, D., Tatomer, D.C., Gold, B., March, Z.M., Cherry, S., and Wilusz, J.E. (2015). Combinatorial control of Drosophila circular RNA expression by intronic repeats, hnRNPs, and SR proteins. Genes Dev. *29*, 2168–2182.

Legnini, I., Di Timoteo, G., Rossi, F., Morlando, M., Briganti, F., Sthandier, O., Fatica, A., Santini, T., Andronache, A., Wade, M., et al. (2017). Circ-ZNF609 Is a Circular RNA that Can Be Translated and Functions in Myogenesis. Mol. Cell *66*, 22–37.e9.

Liang, D., and Wilusz, J.E. (2014). Short intronic repeat sequences facilitate circular RNA production. Genes Dev. *28*, 2233–2247.

Lin, X., and Perrimon, N. (1999). Dally cooperates with Drosophila Frizzled 2 to transduce Wingless signalling. Nature *400*, 281–284.

Lin, Y.J., Seroude, L., and Benzer, S. (1998). Extended life-span and stress resistance in the Drosophila mutant methuselah. Science *282*, 943–946.

Liu, D., Conn, V., Goodall, G.J., and Conn, S.J. (2018). A Highly Efficient Strategy for Overexpressing circRNAs. Methods Mol. Biol. *1724*, 97–105.

Mazin, P., Xiong, J., Liu, X., Yan, Z., Zhang, X., Li, M., He, L., Somel, M., Yuan, Y., Phoebe Chen, Y.P., et al. (2013). Widespread splicing changes in human brain development and aging. Mol. Syst. Biol. *9*, 633.

Memczak, S., Jens, M., Elefsinioti, A., Torti, F., Krueger, J., Rybak, A., Maier, L., Mackowiak, S.D., Gregersen, L.H., Munschauer, M., et al. (2013). Circular RNAs are a large class of animal RNAs with regulatory potency. Nature *495*, 333–338.

Metaxakis, A., Tain, L.S., Grönke, S., Hendrich, O., Hinze, Y., Birras, U., and Partridge, L. (2014). Lowered insulin signalling ameliorates age-related sleep fragmentation in Drosophila. PLoS Biol. *12*, e1001824.

Ni, J.Q., Liu, L.P., Binari, R., Hardy, R., Shim, H.S., Cavallaro, A., Booker, M., Pfeiffer, B.D., Markstein, M., Wang, H., et al. (2009). A Drosophila resource of transgenic RNAi lines for neurogenetics. Genetics *182*, 1089–1100.

Nigro, J.M., Cho, K.R., Fearon, E.R., Kern, S.E., Ruppert, J.M., Oliner, J.D., Kinzler, K.W., and Vogelstein, B. (1991). Scrambled exons. Cell *64*, 607–613.

Pamudurti, N.R., Bartok, O., Jens, M., Ashwal-Fluss, R., Stottmeister, C., Ruhe, L., Hanan, M., Wyler, E., Perez-Hernandez, D., Ramberger, E., et al. (2017). Translation of CircRNAs. Mol. Cell *66*, 9–21.e7.

Pertea, M., Pertea, G.M., Antonescu, C.M., Chang, T.C., Mendell, J.T., and Salzberg, S.L. (2015). StringTie enables improved reconstruction of a transcriptome from RNA-seq reads. Nat. Biotechnol. *33*, 290–295.

Piwecka, M., Glažar, P., Hernandez-Miranda, L.R., Memczak, S., Wolf, S.A., Rybak-Wolf, A., Filipchyk, A., Klironomos, F., Cerda Jara, C.A., Fenske, P., et al. (2017). Loss of a mammalian circular RNA locus causes miRNA deregulation and affects brain function. Science *357*, eaam8526.

Port, F., Chen, H.M., Lee, T., and Bullock, S.L. (2014). Optimized CRISPR/Cas tools for efficient germline and somatic genome engineering in Drosophila. Proc. Natl. Acad. Sci. USA *111*, E2967–E2976.

Reynolds-Peterson, C.E., Zhao, N., Xu, J., Serman, T.M., Xu, J., and Selleck, S.B. (2017). Heparan sulfate proteoglycans regulate autophagy in Drosophila. Autophagy *13*, 1262–1279.

Rodríguez, S.A., Grochová, D., McKenna, T., Borate, B., Trivedi, N.S., Erdos, M.R., and Eriksson, M. (2016). Global genome splicing analysis reveals an increased number of alternatively spliced genes with aging. Aging Cell *15*, 267–278.

Rybak-Wolf, A., Stottmeister, C., Glažar, P., Jens, M., Pino, N., Giusti, S., Hanan, M., Behm, M., Bartok, O., Ashwal-Fluss, R., et al. (2015). Circular RNAs in the Mammalian Brain Are Highly Abundant, Conserved, and Dynamically Expressed. Mol. Cell *58*, 870–885.

Salzman, J., Gawad, C., Wang, P.L., Lacayo, N., and Brown, P.O. (2012). Circular RNAs are the predominant transcript isoform from hundreds of human genes in diverse cell types. PLoS ONE *7*, e30733.

Salzman, J., Chen, R.E., Olsen, M.N., Wang, P.L., and Brown, P.O. (2013). Cell-type specific features of circular RNA expression. PLoS Genet. *9*, e1003777.

Selman, C., Lingard, S., Choudhury, A.I., Batterham, R.L., Claret, M., Clements, M., Ramadani, F., Okkenhaug, K., Schuster, E., Blanc, E., et al. (2008). Evidence for lifespan extension and delayed age-related biomarkers in insulin receptor substrate 1 null mice. FASEB J. *22*, 807–818.

Slack, C., Giannakou, M.E., Foley, A., Goss, M., and Partridge, L. (2011). dFOXO-independent effects of reduced insulin-like signaling in Drosophila. Aging Cell *10*, 735–748.

Starke, S., Jost, I., Rossbach, O., Schneider, T., Schreiner, S., Hung, L.H., and Bindereif, A. (2015). Exon circularization requires canonical splice signals. Cell Rep. *10*, 103–111.

Suzuki, H., Zuo, Y., Wang, J., Zhang, M.Q., Malhotra, A., and Mayeda, A. (2006). Characterization of RNase R-digested cellular RNA source that consists of lariat and circular RNAs from pre-mRNA splicing. Nucleic Acids Res. *34*, e63.

Tain, L.S., Sehlke, R., Jain, C., Chokkalingam, M., Nagaraj, N., Essers, P., Rassner, M., Grönke, S., Froelich, J., Dieterich, C., et al. (2017). A proteomic atlas of insulin signalling reveals tissue-specific mechanisms of longevity assurance. Mol. Syst. Biol. *13*, 939.

Tatomer, D.C., Liang, D., and Wilusz, J.E. (2017). Inducible Expression of Eukaryotic Circular RNAs from Plasmids. Methods Mol. Biol. *1648*, 143–154.

Toyoda, H., Kinoshita-Toyoda, A., Fox, B., and Selleck, S.B. (2000). Structural analysis of glycosaminoglycans in animals bearing mutations in sugarless, sulfateless, and tout-velu. Drosophila homologues of vertebrate genes encoding glycosaminoglycan biosynthetic enzymes. J. Biol. Chem. *275*, 21856–21861.

Trapnell, C., Williams, B.A., Pertea, G., Mortazavi, A., Kwan, G., van Baren, M.J., Salzberg, S.L., Wold, B.J., and Pachter, L. (2010). Transcript assembly and quantification by RNA-Seq reveals unannotated transcripts and isoform switching during cell differentiation. Nat. Biotechnol. *28*, 511–515.

Trapnell, C., Hendrickson, D.G., Sauvageau, M., Goff, L., Rinn, J.L., and Pachter, L. (2013). Differential analysis of gene regulation at transcript resolution with RNA-seq. Nat. Biotechnol. *31*, 46–53.

Wang, P.L., Bao, Y., Yee, M.C., Barrett, S.P., Hogan, G.J., Olsen, M.N., Dinneny, J.R., Brown, P.O., and Salzman, J. (2014). Circular RNA is expressed across the eukaryotic tree of life. PLoS ONE *9*, e90859.

Webber, D.L., Choo, A., Hewson, L.J., Trim, P.J., Snel, M.F., Hopwood, J.J., Richards, R.I., Hemsley, K.M., and O'Keefe, L.V. (2018). Neuronal-specific impairment of heparan sulfate degradation in Drosophila reveals pathogenic mechanisms for Mucopolysaccharidosis type IIIA. Exp. Neurol. *303*, 38–47.

Westholm, J.O., Miura, P., Olson, S., Shenker, S., Joseph, B., Sanfilippo, P., Celniker, S.E., Graveley, B.R., and Lai, E.C. (2014). Genome-wide analysis of Drosophila circular RNAs reveals their structural and sequence properties and age-dependent neural accumulation. Cell Rep. *9*, 1966–1980.

Yamamoto-Hino, M., Abe, M., Shibano, T., Setoguchi, Y., Awano, W., Ueda, R., Okano, H., and Goto, S. (2012). Cisterna-specific localization of glycosylation-related proteins to the Golgi apparatus. Cell Struct. Funct. *37*, 55–63.

Yano, H., Yamamoto-Hino, M., Abe, M., Kuwahara, R., Haraguchi, S., Kusaka, I., Awano, W., Kinoshita-Toyoda, A., Toyoda, H., and Goto, S. (2005). Distinct functional units of the Golgi complex in Drosophila cells. Proc. Natl. Acad. Sci. USA *102*, 13467–13472.

Zhang, Y., You, J., Ren, W., and Lin, X. (2013). Drosophila glypicans Dally and Dally-like are essential regulators for JAK/STAT signaling and Unpaired distribution in eye development. Dev. Biol. *375*, 23–32.

STAR★METHODS

KEY RESOURCES TABLE

REAGENT or RESOURCE	SOURCE	IDENTIFIER
Antibodies		
FLAG M2 (1:10000 diluted)	Sigma-Aldrich	Cat #F1804; RRID:AB_262044
Tubulin (1:5000 diluted)	Sigma-Aldrich	Cat #T9026; RRID:AB_477593
GAPDH (1:1000 diluted)	Santa Cruz	Cat #sc-25778; RRID:AB_10167668
anti-mouse HRP (1:10000 diluted)	Invitrogen	Cat #31430; RRID:AB_228307
anti-rabbit HRP (1:10000 diluted)	Invitrogen	Cat #21234; RRID:AB_2536530
Chemicals, Peptides, and Recombinant Proteins		
Rapamycin	LC Laboratories	Cat #R-5000
Critical Commercial Assays		
Superscript III first-strand synthesis kit	Invitrogen	Cat #18080051
PowerUp SYBR Green Master Mix	Thermo Fisher	Cat #4368706
Pierce BCA Protein Assay Kit	Thermo Fisher Scientific	Cat #23227
Gibson Assembly Cloning Kit	NEB	Cat #E5510S
RNase R	Biozym	Cat #172010
Deposited Data		
RNA seq data	This paper	GEO: GSE130158
Experimental Models: Organisms/Strains		
wDah	Grönke et al., 2010	N/A
da-Gal4	Partridge lab	N/A
elav-Gal4	Partridge lab	N/A
MHC-Gal4	Partridge lab	N/A
NP1-Gal4	Partridge lab	N/A
InsP3-Gal4	Slack et al., 2011	N/A
UAS-foxo	Partridge lab	N/A
UAS-rpr	Slack et al., 2011	N/A
dFoxoΔ94	Slack et al., 2011	N/A
dilp 2-3,5	Grönke et al., 2010	N/A
pValium Sfl RNAi	Bloomington	Cat #34601
UAS-Sfl-HA	Yamamoto-Hino et al., 2012	N/A
UAS-dally	Bloomington	Cat #5397
UAS-dally-like	Bloomington	Cat #9160
UAS-syndecan	Bloomington	Cat #8564
y^1w^1; vas-phiC31; attP40	Bischof et al., 2007	N/A
y^1v^1; nanos-phiC31; attP40	Bloomington	Cat #25709
nanos-Cas9	Bloomington	Cat #54591
UAS-dally RNAi	VDRC	v14136
mth1	Bloomington	27896
UAS-circBtsz-exon	This paper	N/A
UAS-circBtsz-1000	This paper	N/A
UAS-circBtsz-inv	This paper	N/A
pWalium20-circBtsz-1	This paper	N/A
UAS-circSfl-exon	This paper	N/A
UAS-circSfl-1000	This paper	N/A

(Continued on next page)

CellPress
OPEN ACCESS

Continued

REAGENT or RESOURCE	SOURCE	IDENTIFIER
UAS-circSfl-inv	This paper	N/A
UAS-FLAG::circSfl-inv	This paper	N/A
pCFD4 Sfl N term	This paper	N/A
FLAG::Sfl	This paper	N/A
pWalium20-circSfl-1	This paper	N/A
pWalium20-circSfl-2	This paper	N/A
pWalium20-circSfl-3	This paper	N/A
pCFD4 Sfl^{ex2}	This paper	N/A
$sfl^{\Delta ex2}$	This paper	N/A
UAS-circSfl-ORF	This paper	N/A
Oligonucleotides		
See Tables S1 and S3	N/A	N/A
Recombinant DNA		
BAC clone *sfl* gene	PACMAN	CH321-22A12
BAC clone *btsz* gene	PACMAN	CH321-61F02
pUAST attb-circBtsz-exon	This paper	N/A
pUAST attb circBtsz-1000	This paper	N/A
pUAST attb circBtsz-inv	This paper	N/A
pWalium20 circBtsz-1	This paper	N/A
pUAST attb-circSfl-exon	This paper	N/A
pUAST attb-circSfl-1000	This paper	N/A
pUAST attb-circSfl-inv	This paper	N/A
pUAST attb FLAG::circSfl-inv	This paper	N/A
pCFD4 Sfl N Term	This paper	N/A
pBS FLAG::Sfl Donor	This paper	N/A
pWalium20-circSfl-1	This paper	N/A
pWalium20-circSfl-2	This paper	N/A
pWalium20-circSfl-3	This paper	N/A
pCFD4 $sfl^{\Delta ex2}$	This paper	N/A
pUAST-attb-circSfl-ORF	This paper	N/A
Software and Algorithms		
DCC	Cheng et al., 2016	N/A
Microsoft Excel	Microsoft	N/A
GraphPad Prism	GraphPad	https://www.graphpad.com/scientific-software/prism/
FIMO	The MEME Suite	http://meme-suite.org/tools/fimo
Other		
Criterion TGX Stain-Free Precast Gels, any kD	BioRad	Cat #5678123

RESOURCE AVAILABILITY

Lead Contact

Further information and requests for resources and reagents should be directed to and will be fulfilled by the Lead Contact, Linda Partridge (partridge@age.mpg.de).

Materials Availability

Materials such as transgenic fly lines or plasmids will be available without further restrictions upon request to the Lead Contact (partridge@age.mpg.de).

Data and Code Availability

The tissue-specific RNA sequencing of young, middle-aged and old-age flies was deposited in NCBI's Gene Expression Omnibus (Edgar et al., 2002). The accession number for the RNA sequencing data reported in this paper is GEO: GSE130158.

EXPERIMENTAL MODEL AND SUBJECT DETAILS

Fly husbandry

Fly stocks were kept at 25°C on a 12 h light and 12 h dark cycle and fed a sugar/yeast/agar diet (Bass et al., 2007). In all experiments, females and males were reared at controlled larval densities and allowed to mate for 48h (''once-mated'') before each experiment. If not indicated otherwise, female flies were used for all experiments. Flies were snap frozen with liquid nitrogen. Dissections were carried out in phosphate-buffered saline (PBS) and tissues were frozen in dry ice. Rapamycin food was prepared by adding Rapamycin to a concentration of 200 μM. Control food contained the same volume of the carrier EtOH (Bjedov et al., 2010). The outbred wild-type strain *white Dahomey* (*wDah*) (Grönke et al., 2010) that naturally carries the endosymbiontic bacterium *Wolbachia* was used for all experiments and maintained in large population cages with overlapping generations. All mutations and transgenic constructs were backcrossed into the white Dahomey (wDah) wild-type background for at least six generations.

METHOD DETAILS

Transgenic flies

The transgenic fly lines used in this study are shown in the Key Resources Table.

Lifespan analysis

Flies were reared at controlled larval density. Once-mated flies were anaesthetized by CO_2, sorted by sex and transferred to vials (10 flies/vial). Three times a week, flies were transferred to fresh vials and deaths were scored. Standard SYA (sugar-yeast-agar) food was used throughout. 15-20 replicates (= 150 – 200 flies in total) were used per condition. Female flies were used for all lifespan experiments if not indicated otherwise.

Fecundity assay

During lifespan experiments, fecundity was analyzed on day 7. Flies were transferred to fresh vials in the afternoon at around 5 pm. Each vial contained 10 flies. The next morning (16 h later), eggs were counted in 10 vials per condition. Egg laying was calculated for 24 h and the mean number of eggs is reported.

Stress assays

20 once-mated flies per vial were transferred to standard food and aged for 7 days. Stress assays were started on day 7 with different diets: Starvation assay (1% agarose in ddH_2O) or Oxidative stress (5% H_2O_2, 5% sucrose, 1% agar in ddH_2O). Deaths were scored 2-3 times a day. 5 replicates (n = 100 flies) were used per condition.

Development assay

25 eggs were manually placed in a food vial containing SYA medium and incubated at 25°C. 10 replicates (n = 250 eggs) per condition were used. Flies eclosing were scored twice a day to measure development time. Female flies were used for analysis. For each vial, number of eclosing females was scored and divided by the total number of eclosed females in each vial to calculate the mean eclosion day for each vial.

Climbing assay

Flies were placed half an hour prior to measurements in the Climbing assay plastic tubes for acclimation. Subsequently, flies were transferred to vial 1 of the six-compartment climbing apparatus (Greene et al., 2003) and tapped down to the bottom of the vial. Flies could climb to the upper vial ranging a distance of 15 cm for 20 s. Flies in the upper vial were transferred into vial 2 and tapped down again. This procedure was repeated 5 times. The climbing index (CI) was calculated based on the number of flies in each vial (lowest = 0 = all flies in vial 1, highest = 1 = all flies in vial 5). 20 flies per tube and 3 biological replicates were used per condition.

Molecular Cloning

All restriction digest reactions were performed with enzymes provided by NEB according to their user's manual. T4 DNA Ligase (NEB) was used for ligation reactions. If necessary, plasmids were modified with the QuikChange II Site-Directed Mutagenesis Kit (Agilent). pCFD4 cloning for CRISPR was conducted according to Port et al. (2014). In brief, oligonucleotides including guideRNA sequences were introduced into the pCFD4 vector by PCR followed by Gibson assembly. Positive clones were identified by Sanger sequencing. pWalium20 cloning for siRNA-mediated knock-down constructs was conducted according to the TRiP protocol (Ni et al., 2009). In brief, oligonucleotides including the siRNA sequence and overhanging restriction sites were annealed and ligated into a pre-digested pWalium20 vector. Positive clones were identified by Sanger sequencing. Transgenic fly lines were generated using

CellPress
OPEN ACCESS

CRISPR/Cas9-mediated genomic engineering (Port et al., 2014) or the φC31 and attP/attB integration system (Bischof et al., 2007) using the attP40 landing-site. Embryos were injected with 400 ng/μL DNA at the Max Planck Institute for Biology of Aging. For CRISPR-mediated knock-in, nanos-Cas9 expressing flies were crossed with pCFD4 guideRNA expressing flies. The progeny were injected with the appropriate donor template and positive CRISPR events were identified by PCR and sequencing. Cloning strategies and primers used are described in Table S1. Transgenic flies generated in this study are summarized in Table S2.

RNA extraction, cDNA synthesis and q-RT-PCR

Total RNA was extracted using Trizol (Invitrogen) according to the manufacturer's instructions, including a DNase (QIAGEN) treatment. cDNA of mRNA and circRNAs was generated using the SuperScript III first-strand synthesis kit (Invitrogen) using random hexamers. 600 ng total RNA was used for cDNA synthesis. For q-RT-PCR of mRNA or circRNAs PowerUp SYBR Green Master Mix (ThermoFisher) was used according to the manufacturer's manual. q-RT-PCR was performed with a 7900HT real-time PCR system (Applied Biosystems) or with a QuantStudio7 (ThermoFisher). Relative expression (fold induction) was calculated using the ΔΔCT method and Rpl32 as normalization control. Primer sequences used for q-RT-PCR are shown in Table S3.

RNA sequencing

Flies were reared under controlled larval densities and aged until 10, 30 or 50 days. At each time point, adult fly tissues were manually dissected from female flies: brain, thorax (without gut), gut (without crop but with malpighian tubules), fat body (abdomen without the gut and without ovaries). Dissected tissues were immediately transferred to a tube on dry ice. 3 biological replicates were used per condition. Notably, day 10 samples were generated independently. Total RNA was extracted using Trizol (Invitrogen) according to the manufacturer's instructions, including DNase treatment. Quality control was performed by Experion Automated Electrophoresis System (Biorad). Three biological replicates were used per condition. rRNA depleted libraries were generated at the Max Planck-Genome-Centre Cologne (Germany). RNA sequencing was performed with an Illumina HighSeq2500 and ~37.5 million single-end reads/sample. For circRNA detection, raw reads were mapped to the *Drosophila* genome BDGP6 using STAR version 2.5.2b. CircRNAs were detected using DCC version 0.4.4 (Cheng et al., 2016). CircRNAs were annotated, if 5 reads were detected in at least 6 samples. Differential expression of circRNAs was tested by beta-binomial test (bb.test from R package "ibb"). CircRNAs were normalized to the linear spliced reads of their own host genes. For tissue-specific annotation of circRNAs, circRNAs that were detected in a tissue in at least one of the samples and at least one read were considered. For isoform-specific linear transcript analysis, raw reads were mapped to BDGP6 using HISAT version 2.0.4 (Kim et al., 2015) and transcripts were assembled with StringTie version 1.3.0 (Pertea et al., 2015). Transcripts were quantified, normalized and analyzed using Cufflinks version 2.2.1 (Trapnell et al., 2010, 2013).

circRNA sequence analysis

MEME Suite's FIMO tool (Grant et al., 2011) was used to detect miRNA sequences within the circRNA sequence using "Drosophila_melanogaster_dme.dna_encoded" database. Stop codon conservation within circSfl was manually curated using UCSC genome browser (Kent et al., 2002).

RNase R treatment

1 μg of total RNA was incubated with 3 U RNase R (Biozym) at 37°C for 15 min. After isopropanol precipitation and washes with EtOH, the same volume of digested sample and mock treated sample was used for standard cDNA synthesis and q-RT-PCR.

Polysome profiling

Polysome profiling was performed as previously described with minor modifications (Dinkova et al., 2005). 300 μL of ice-cold polysome buffer (300 mM NaCl, 50 mM Tris-HCL (pH 8.0), 10 mM MgCl2, 1 mM, EGTA, 200 mg heparin/ml, 400 U RNAsin/ml, 1.0 mM, phenylmethylsulfonyl fluoride, 0.2mg cycloheximide/ml, 1% Triton X-100, 0.1% Sodium deoxycholate) were added to frozen tissues and homogenized for up to 10 s with a hand gun on ice. 700 μL polysome buffer were added, mixed and samples were incubated on ice for 10 min. To clarify lysate, samples were centrifuged at 13000 rpm and 4°C for 10 mins. Around 800 μL of supernatant were removed while avoiding the pellet and potential fat layer on top. Nucleic acid content of samples was measured by NanoDrop recording A260 units for normalization (5-10 units). 700 μL sample were layered on top of the 10%–50% sucrose in high salt resolving buffer (140 mM NaCl, 25 mM Tris-HCL (pH 8.0), 10 mM $MgCl_2$) gradient and spun for 1.5 h at 38000 rpm using an Beckman SW41Ti rotor. After running the blank gradient (with 700 μL polysome buffer without sample), sample gradients were run. Samples were then collected dropwise as monosome fraction and polysome fraction. Profiles were monitored (Ab 252 nm) using a Teledyne density gradient fractionator. Total RNA from the polysome fraction was extracted using Trizol (Invitrogen) according to the manufacturer's instructions, including DNase treatment (QIAGEN). Quality control was performed by Experion Automated Electrophoresis System (Biorad). Three biological replicates were used per condition. rRNA depleted libraries were generated at the Max Planck-Genome-Centre Cologne (Germany). RNA sequencing was performed with an Illumina HighSeq2500 and ~37.5 million 100 bp single-end reads/sample. Bioinformatic analysis was performed similar to the total RNA sequencing data (see above), but circRNA reads were normalized to total RNA counts.

Western blotting

Proteins of mechanically separated heads (25 per replicate) were extracted with RIPA buffer (Pierce) supplemented with Complete mini protease inhibitor without EDTA (Roche). Proteins were quantified using BCA Protein Assay Kit (ThermoFisher), and 30 μg of protein was loaded on pre-stained SDS–PAGE gels (Bio-Rad). Proteins were transferred to 0.45 μm Nitrocellulose Membranes (GE Healthcare) using wet transfer for 30 min. Unspecific binding was blocked using 5% non-fat dry milk powder in TBST. Primary antibodies were diluted (see Key Resources Table) and incubated with the membrane over night at 4°C. HRP-coupled secondary antibodies (ThermoFisher) were used according to the primary antibody. Signal was developed using ECL Western Blotting Detection Reagents (GE Healthcare) and the ChemiDocImager (BioRad). To allow robust quantification, exposure time of the blots was adjusted to the expression level of each protein individually. Tubulin or GAPDH were used as normalization control. Antibodies used in this study are shown in the Key resource table.

QUANTIFICATION AND STATISTICAL ANALYSIS

Statistical analysis was performed using GraphPad Prism and R. Individual statistical tests are mentioned in the respective figure legends. 1-way ANOVA was followed by Tukey post hoc test. 2-way ANOVA was always followed by Bonferroni post hoc test. Life-span assays were recorded using Excel and survivorship was analyzed using log rank test and Cox proportional hazard analysis. Bar plot graphs represent the mean values ± SEM. The number of biological replicates (n) is stated in each figure legend. The number of flies in each biological replicate is depending on the method used and stated in the STAR Methods part. Significance was determined according to the p value: *$p < 0.05$, **$p < 0.01$, ***$p < 0.001$, ****$p < 0.0001$.

Molecular Cell, Volume *79*

Supplemental Information

An Insulin-Sensitive Circular RNA that Regulates Lifespan in *Drosophila*

Carina Marianne Weigelt, Rohan Sehgal, Luke Stephen Tain, Jun Cheng, Jacqueline Eßer, André Pahl, Christoph Dieterich, Sebastian Grönke, and Linda Partridge

Supplemental Figures

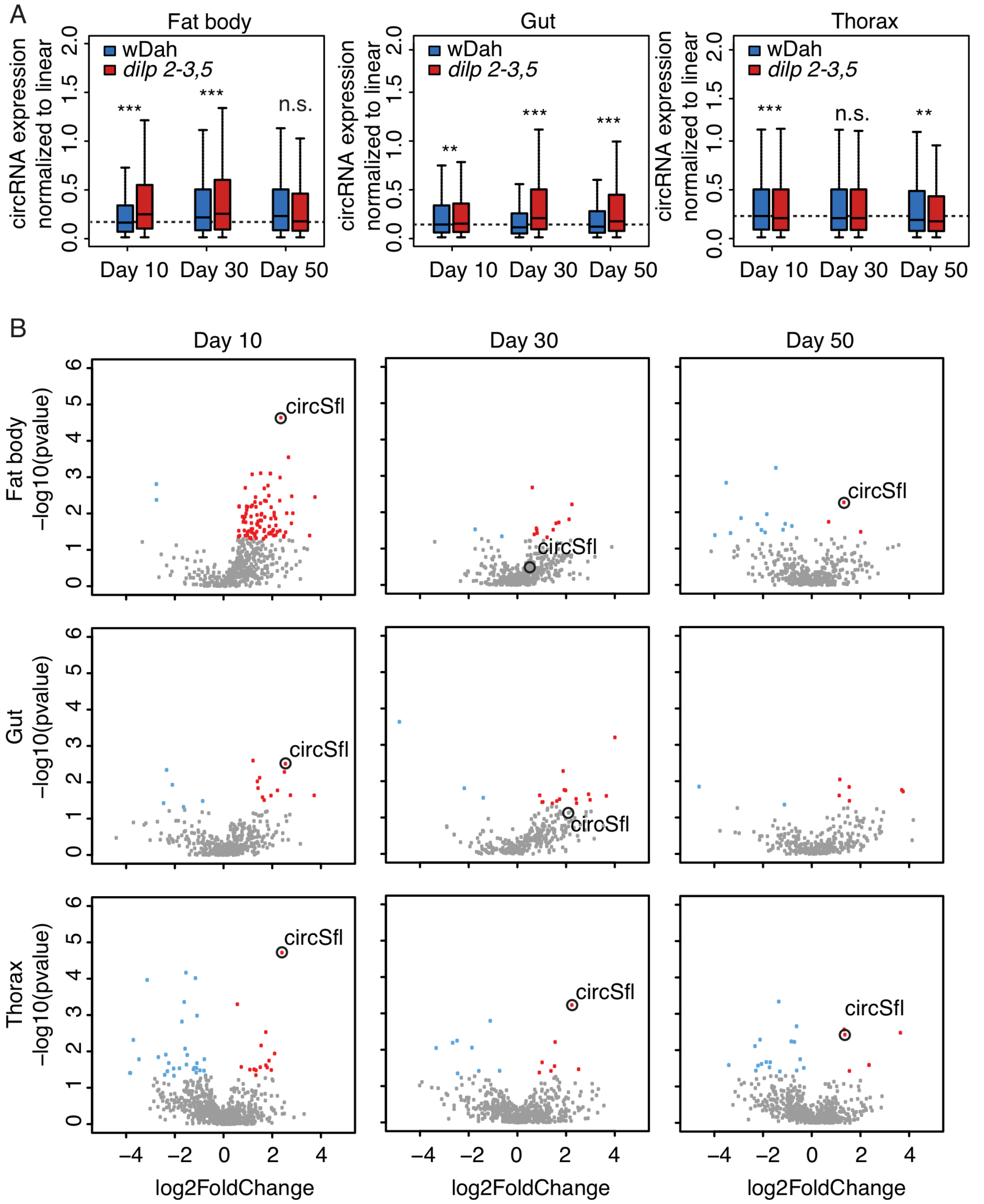

Figure S1: Differentially expressed circRNAs in the fat body, gut and thorax of dilp *2-3,5* mutant flies, Related to Figure 1. (A) Global circRNA expression was only mildly increased during ageing in the fat body, unchanged in the gut and mildly decreased in the thorax of *wDah* wildtype flies. Global circRNA levels were up-regulated in fat body and gut of *dilp 2-3,5* mutants (fat body: genotype p<0.05, interaction with age: p<0.0001; gut: genotype: p<0.0001, interaction with age: p<0.0001, 2-way ANOVA, n=3) and not significantly changed in the thorax of *dilp2-3,5* mutants (n=3). (B) CircSfl was up-regulated in most tissues and at all ages in *dilp2-3,5* mutants (p<0.05, beta-binomial test, n=3).

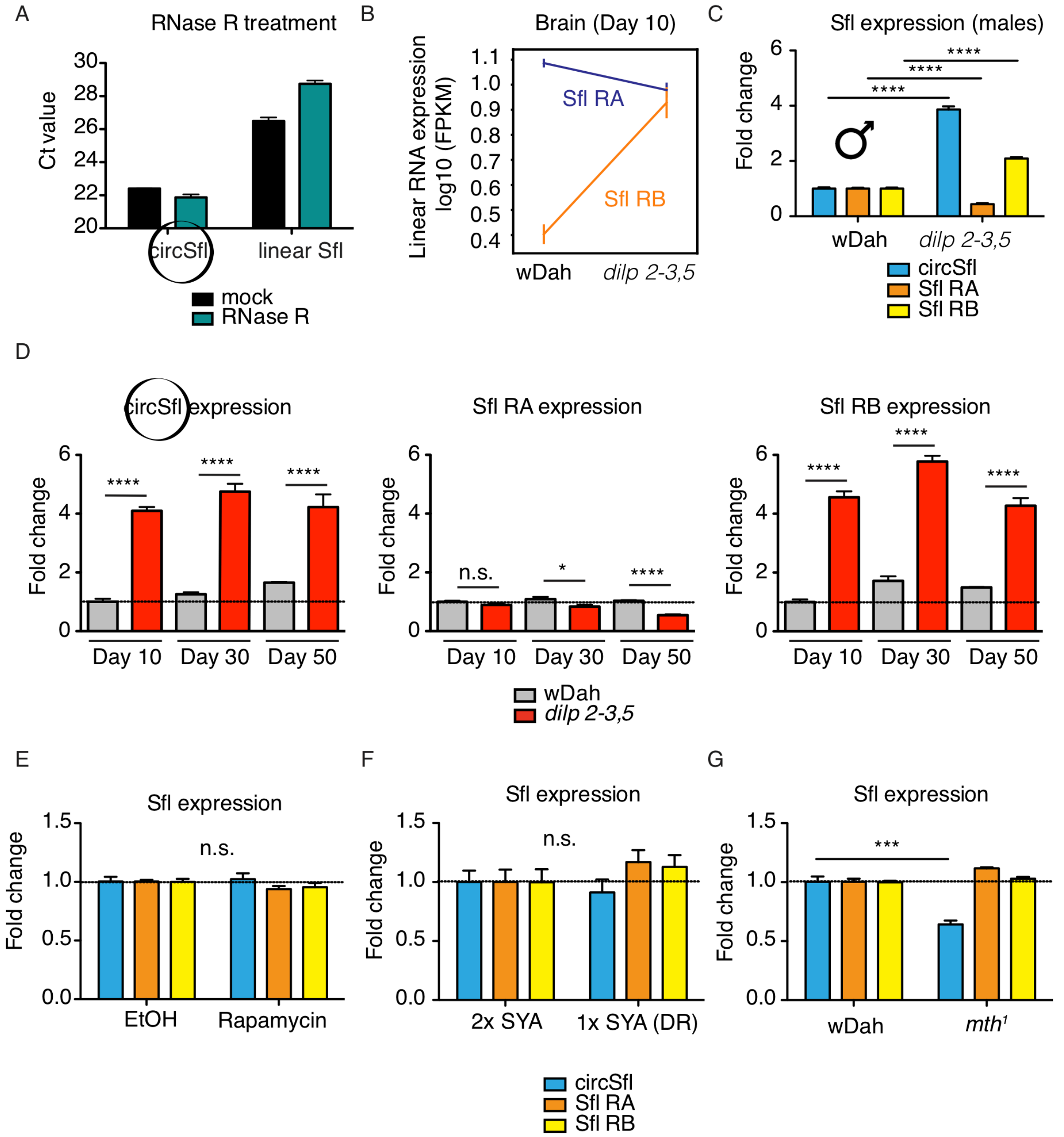

Figure S2: Verification and regulation of circSfl, Related to Figure 2. (A) RNase R treatment did not affect circSfl stability, but degradated linear Sfl transcripts as indicated by higher Ct values as measured by q-RT-PCR and normalized to input. (B) RNA sequencing on brains showed that Sfl RA was the predominantly expressed linear isoform in wildtype flies. In contrast, *dilp 2-3,5* mutants expressed Sfl RA and RB at similar levels. (C) CircSfl was also up-regulated in male *dilp 2-3,5* mutants (whole flies), while Sfl RA was decreased and Sfl RB increased compared to wDah control flies (Student's t-test, n = 4). (D) CircSfl was up-regulated in young, middle-aged and old *dilp2-3,5* mutant fly heads. While Sfl RA transcript levels were not increased in insulin mutant flies, Sfl RB transcript levels were increased at all investigated timepoints (*p<0.05, ***p<0.001, ****p<0.0001, 2-way ANOVA with Bonferroni post-hoc test). (E) Reduction of TOR signalling by application of rapamycin did

not affect circSfl or linear Sfl expression levels in fly heads (Student's t-test, n=4). (F) Dietary restriction (1x SYA) did not affect transcript levels of circSfl, Sfl RA or Sfl RB in comparison to control fully fed female flies (2x SYA) (Student's t-test, n = 4). (G) CircSfl expression was decreased in *mth*^{1} mutant female flies (Student's t-test, n = 4).

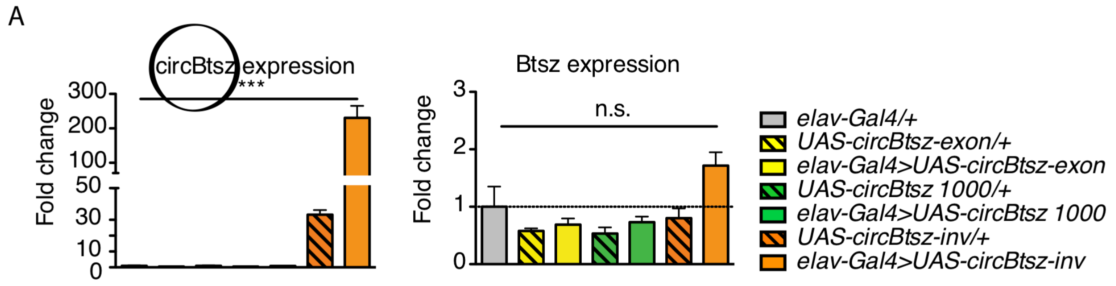

Figure S3: Generation of circBtsz over-expression mutants, Related to Figure 3. (A) CircBtsz was strongly over-expressed in *elav-Gal4>UAS-circBtsz-inv* flies, but not in the other transgenic lines tested (**p<0.01, ***p<0.001, 1-way ANOVA with Tukey post-hoc test, n=3).

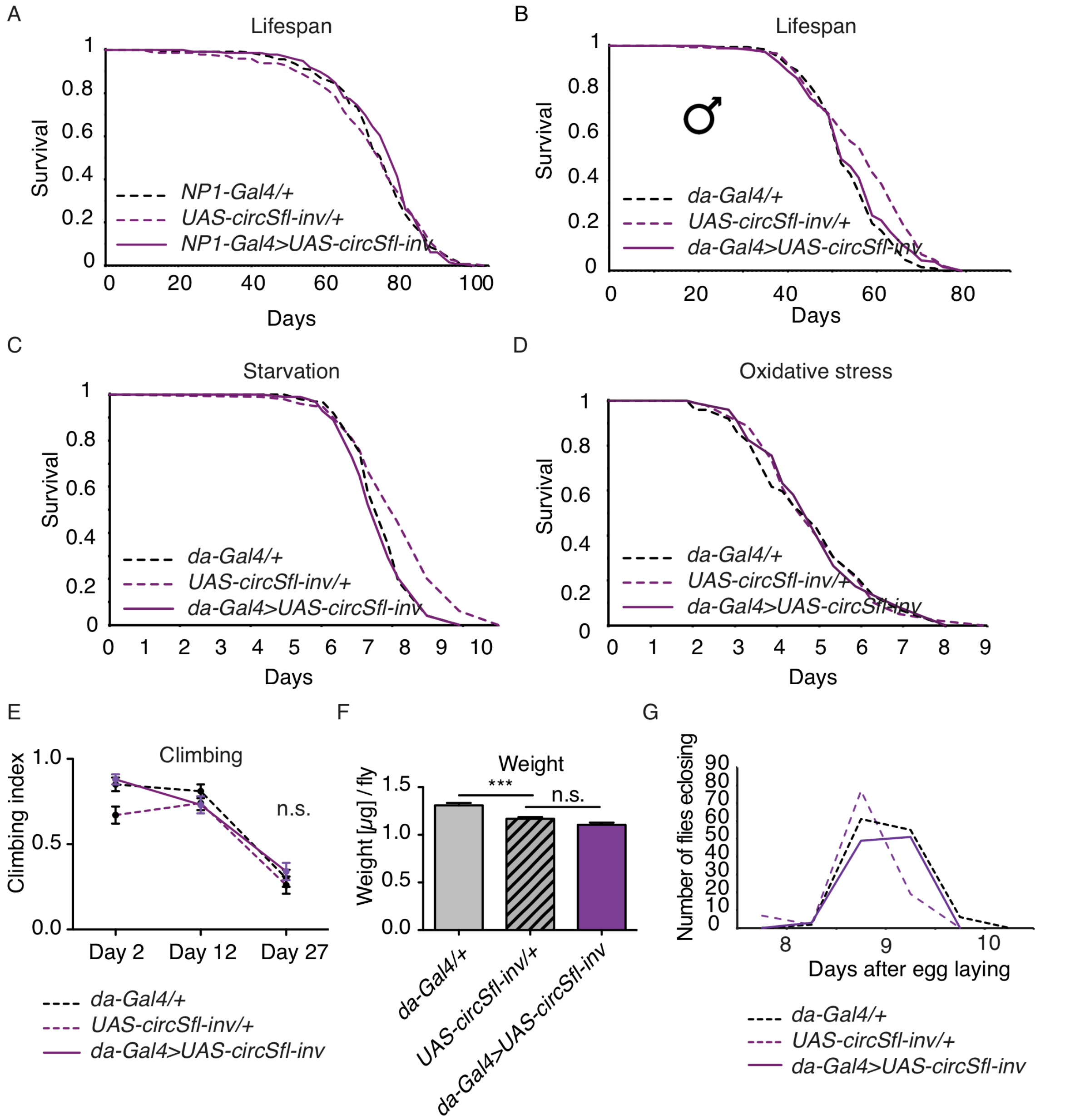

Figure S4: Overexpression of circSfl does not affect stress resistance, body weight or development, Related to Figure 3. (A) Gut-specific over-expression of circSfl by NP1-Gal4 did not affect female lifespan. (B) Ubiquitous over-expression of circSfl did not affect lifespan of male flies. **(**C-D) CircSfl over-expression mutants showed a normal response to (C) starvation (n~100) and (D) to oxidative stress (log-rank test, n~100). (E) CircSfl over-expression mutants showed normal climbing abilities (2-way ANOVA, n~60). (F) Body weight of circSfl over-expression mutants was not different to the *UAS-circSfl/+* control (1-way ANOVA with Tukey post-hoc test, n=10). (G) Mean development time of circSfl over-expression mutants was not different compared to the *da-Gal4/+* control (1-way ANOVA).

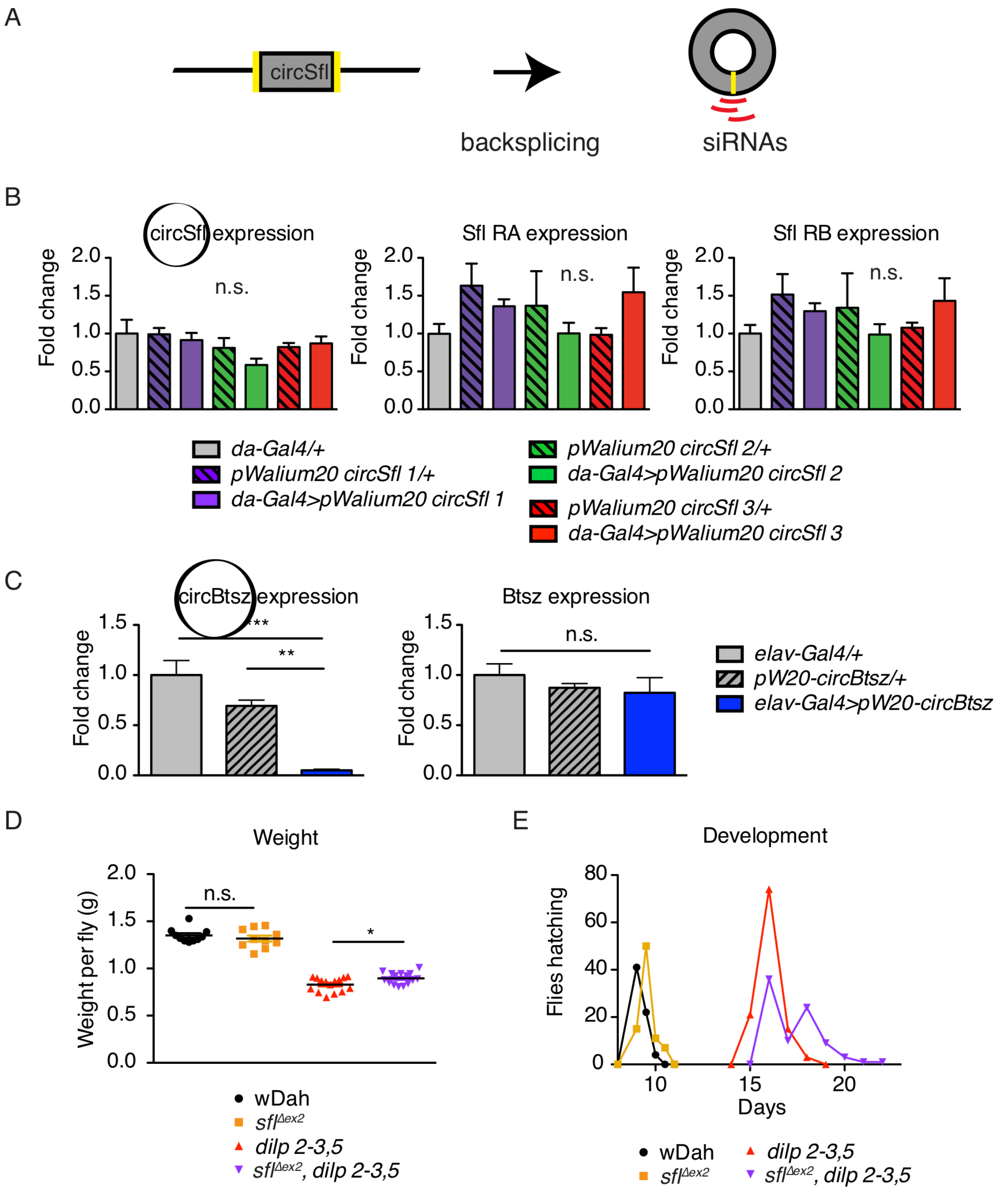

Figure S5: SiRNA mutants to knock-down circRNAs and weight and development of *sfl*$^{\Delta ex2}$ mutants, Related to Figure 4. (A) Three different siRNAs were used to target the circRNA-specific backsplice junction. (B) circSfl expression levels were not significantly decreased in fly lines expressing siRNAs against the circSfl-specific splice junction. (C) CircBtsz expression was strongly reduced in flies expressing siRNAs targeting the circBtsz-specific backsplice junction, but not the linear Btsz transcript. (D) Weight of *sfl*$^{\Delta ex2}$ mutants was similar to wDah control flies. The reduced weight of *dilp 2-3,5* mutants was partially rescued in *sfl*$^{\Delta ex2}$*, dilp 2-3,5* double mutants (*sfl*$^{\Delta ex2}$: n.s., *dilp 2-3,5*: ****p<0.0001, interaction: *p<0.05, 2-way ANOVA with Bonferroni post-hoc test, n = 10-15). (E) *sfl*$^{\Delta ex2}$*, dilp 2-3,5* double mutants were even more developmentally delayed as *dilp 2-3,5* mutants (Mean

eclosion day: *sfl*$^{\Delta ex2}$, *dilp 2-3,5* vs. *dilp 2-3,5:* p<0.0001, interaction between *sfl*$^{\Delta ex2}$ and *dilp 2-3,5: p<0.0001,* 2-way ANOVA with Bonferroni post-hoc test, n ~ 60-130).

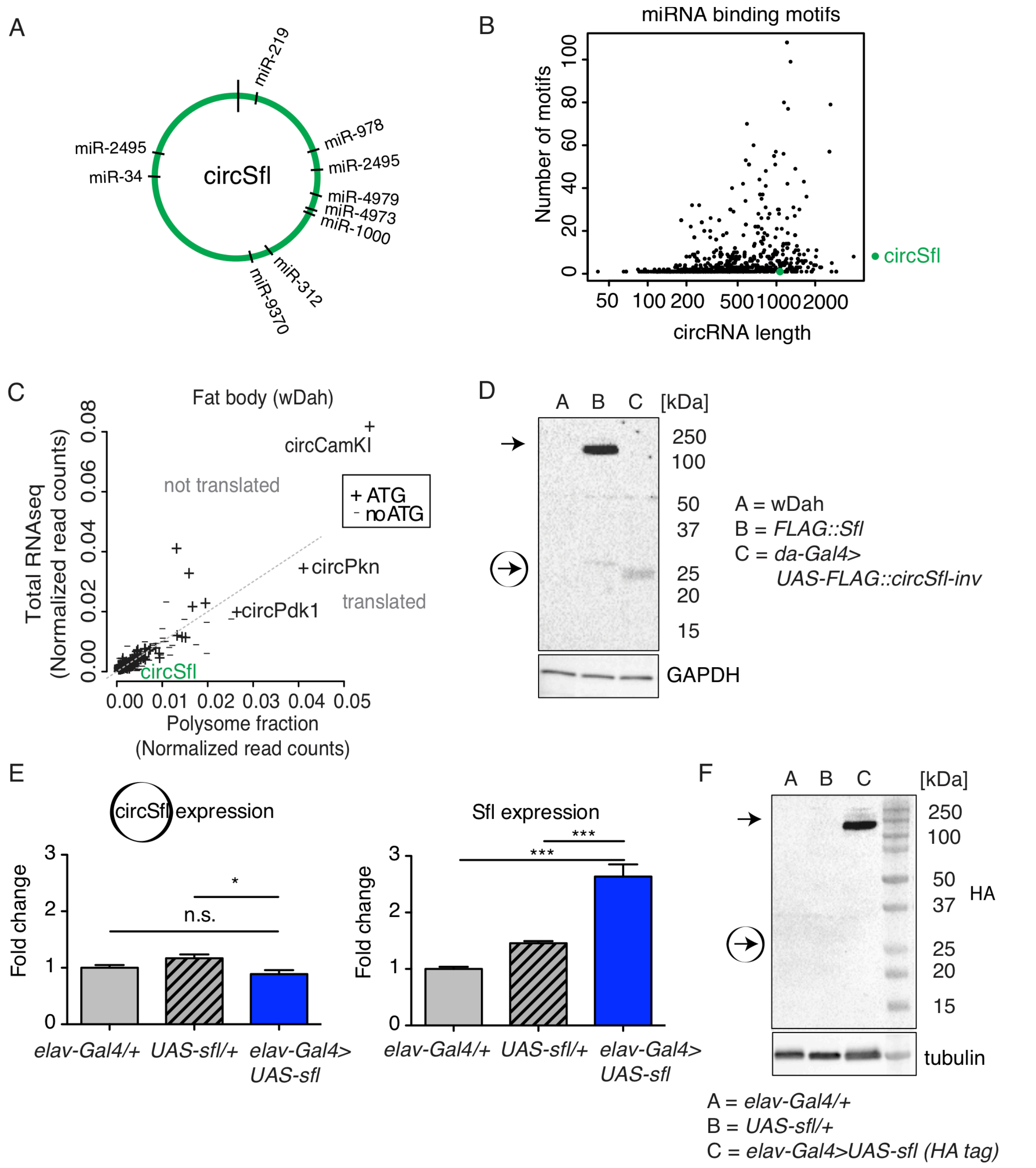

Figure S6: The 25kDa Sfl protein isoform does result from proteolytic cleavage of the full length Sfl protein, Related to Figure 5. (A) 10 different miRNA seed sequences were identified in the circSfl transcript. (B) Number of miRNA binding motifs in circSfl (green) compared to all other circRNAs in *Drosophila*. Note, the number of identified miRNA binding sites in circSfl is lower in this analysis compared to (A), as all circRNs were analysed in parallel, which reduces the power to detect individual sites. (C) Low levels of circSfl were detected by polysome-profiling in wildtype flies indicative for low levels of circSfl translation.

(D) Over-expression of FLAG::circSfl-inv resulted in a single band of around 25 kDa similar to the smaller band observed in *FLAG::Sfl* knock-in flies. (E) Over-expression of an HA-tagged Sfl cDNA *(elav-Gal4>UAS-sfl)* increased linear Sfl transcript levels but not circSfl levels (***p<0.001, *p<0.05, 1-way ANOVA with Tukey post-hoc test, n =3). (F) Western blot analysis identified a single band of around 110 kDa corresponding to the full-length Sfl protein in HA-tag Sfl cDNA over-expressor flies. No protein was detected at the size of 25kDa.

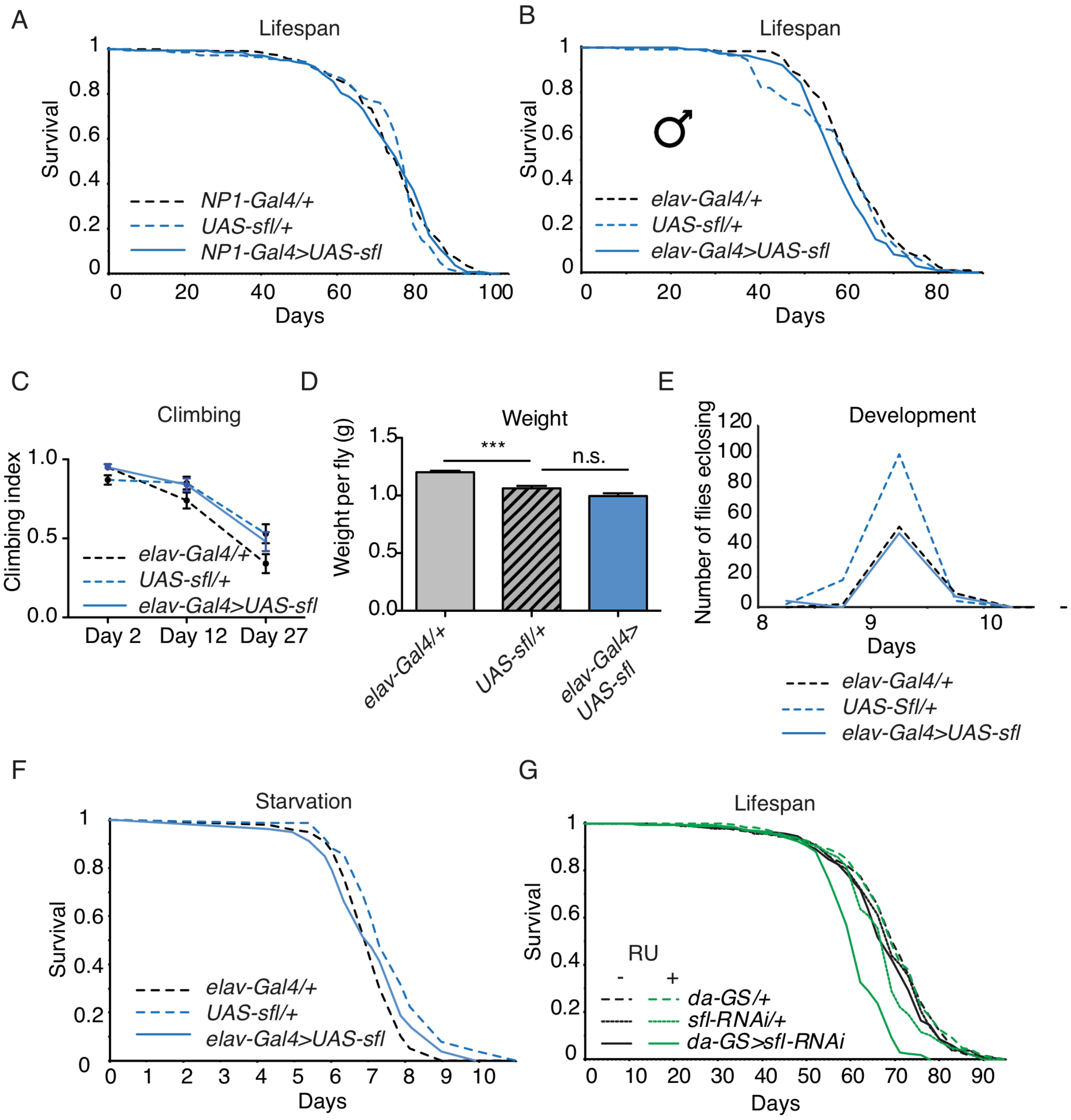

Figure S7: Sfl over-expression mutants have a normal starvation response, climbing ability, weight and development, Related to Figure 6. (A) Over-expression of Sfl in the gut by NP1-Gal4 did not affect lifespan of female flies. (B) Neuron-specific over-expression of

Sfl did not affect lifespan of male flies. (C) Female Sfl over-expression (*elav-Gal4>UAS-sfl*) mutants showed normal climbing ability. (D) The body weight of Sfl over-expression mutants was not different to *UAS-sfl/+* controls (1-way ANOVA with Tukey post-hoc test, n=10). (E) Development time of Sfl over-expression mutants was not changed compared to both controls (1-way ANOVA). (F) Over-expression of Sfl in neurons did not alter starvation resistance. (G) Adult-onset knock-down of Sfl by RU induction shortened lifespan (*da-GS>sfl RNAi* uninduced vs. induced: p<0.0001, log rank test, n~200).

Supplemental Tables

Table S1: Cloning strategy for vectors generated in this study including primers used., Related to STAR Methods. Underlined bases highlight the restriction site used. **Fat** bases highlight siRNA or guideRNA sequences within the primers.

Vector name	Vector backbone	Primer names	Primer sequences	Method
pUAST attb-circBtsz-exon	pUAST attb	CW113	ATGAATTCGTATCAATTTGCCCACCGGCAGG	Restriction digest via EcoRI and NotI (EcoRI is naturally present)
		CW114	ATGCGGCCGCATCATTCTAGTGGCATCTCTCTGCC	
pUAST attb circBtsz-1000	pUAST attb	CW102	AGTGAATTCTCATTGAATTGGCTTTGGA	Restriction digest via EcoRI and NotI (EcoRI is naturally present)
		CW103	ATGCGGCCGCGGCAACAATTTTCCGCGCATATCA	
pUAST attb circBtsz-inv	pUAST attb	CW102	AGTGAATTCTCATTGAATTGGCTTTGGA	1) Restriction digest via EcoRI + NotI (CW102 + 192)<br>2) Restriction digest via NotI (CW193 + 194)
		CW192	ATGCGGCCGCCGTTCGGTCGATATATGTGGCTCTAA	
		CW193	ATGCGGCCGCTCATTGAATTGGCTTTGGA	
		CW194	ATGCGGCCGCGCCCCTACAATTCCCTGTTCATATTT	
pWalium20 circBtsz-1	pWalium 20	CW168	CTAGCAGT**CACTAGAATGATGTATCAATT**TAGTTATATTCAAGCATA**AATTGATACATCATTCTAGTG**GCG	pWalium cloning<br><br>Ni et al. (2008)
		CW169	AATTCGC**CACTAGAATGATGTATCAATT**TATGCTTGAATATAACTA**AATTGATACATCATTCTAGTG**ACTG	
		CW187	ATGAGCTCTTTAATTAGGAAAGCAAGGGAGAGGCGG	
pUAST	pUAST	CW090	ATGCGGCCGCATAAGAGG	Restriction digest

attb-circSfl-exon	attb		ACGACCCACTGGGACG	with NotI and KpnI
		CW091	ATGGTACCCCATTAAACACGCCCGTATCGACAT	
pUAST attb-circSfl-1000	pUAST attb	CW085	ATGCGGCCGCCAAACATTGTTGGCCATCTAAAACT	Restriction digest via NotI and KpnI
		CW086	ATGGTACCGCTTGCACCAAATGAGTTTTATTCT	
pUAST attb-circSfl-inv	pUAST attb	CW085	ATGCGGCCGCCAAACATTGTTGGCCATCTAAAACT	1) Restriction digest via NotI and KpnI (CW085 + 088)<br>2) Restriction digest via KpnI (CW087 + CW089)
		CW088	ATGGTACCTGTCTCCCACATAGTTTCAAATCCCA	
		CW087	ATGGTACCCAAACATTGTTGGCCATCTAAAACT	
		CW089	ATGGTACCTCTAATCAGGTCAAAAGTTGTTAGCCT	
pUAST attb FLAG::circSfl-inv	pUAST attb-circSfl-inv	Gene Synthesis	Add FLAG tag before circSfl ORF	1) Restriction digest via NotI and KpnI<br>2) Subclone in pBS SK<br>3) Insert GeneSynthesis product via XbaI + NsiI<br>4) Clone into pUAST attb via NotI + KpnI<br>5) Add intron via KpnI
pCFD4 Sfl N Term	pCFD4	CW224	TATATAGGAAAGATATCCGGGTGAACTTC**GTCGCTCTGTTTAGTCAATAT**GTTTTAGAGCTAGAAATAGCAAG	pCFD4 Cloning<br><br>Port et al. (2014)
		CW225	ATTTTAACTTGCTATTTCTAGCTCTAAAAC**TGACCGGCACCCAAACGGAT**CGACGTTAAATTGAAAATAGGTC	
pBS FLAG::Sfl Donor	pBluescript SK+	CW265	ATGGTACCACCGAAATGCCACCATCAATTAGTT	1) Restriction Digest via KpnI + NotI<br>2) Insert Gene Synthesis Product via EcoRI and HindIII
		CW266	ATGCGGCCGCAAACCGAGCAGACTGAAGTGCATTT	
pWalium20-circSfl-1	pWalium 20	CW164	CTAGCAGT**GTGTTTAATGGATAAGAGGAC**TAGTTATATTCAAGCATA**GTCCTCTTATCCATTAAACAC**GCG	pWalium cloning<br><br>Ni et al. (2008)
		CW165	AATTCGC**GTGTTTAATGGATAAGAGGAC**TATGCTTGAATATAACTA**GTCCTCTTATCCATTAAACAC**ACTG	
pWalium20-circSfl-2	pWalium 20	CW360	CTAGCAGT**GCGTGTTTAATGGATAAGAGG**TAGTTATATTCAAGCATA**CCTCTTA**	pWalium cloning<br><br>Ni et al. (2008)

			**TCCATTAAACACGC**GCG	
		CW361	AATTCGC**GCGTGTTTAATGGATAAGAGG**TATGCTTGAATATAACTA**CCTCTTATCCATTAAACACGC**ACTG	
pWalium20-circSfl-3	pWalium 20	CW362	CTAGCAGT**GTTTAATGGATAAGAGGACGA**TAGTTATATTCAAGCATA**TCGTCCTCTTATCCATTAAAC**GCG	pWalium cloning<br><br>Ni et al. (2008)
		CW363	AATTCGC**GTTTAATGGATAAGAGGACGA**TATGCTTGAATATAACTA**TCGTCCTCTTATCCATTAAAC**ACTG	
pCFD4 $sfl^{\Delta ex2}$	pCFD4	CW184	TATATAGGAAAGATATCCGGGTGAACTTC**GACCAGCTTGCGGAGATTCGC**GTTTTAGAGCTAGAAATAGCAAG	pCFD4 Cloning<br><br>Port et al. (2014)
		CW185	ATTTTAACTTGCTATTTCTAGCTCTAAAAC**AATTTTATCCAGTAATGAAAC**GACGTTAAATTGAAAATAGGTC	
pUAST-attb-circSfl-ORF	pUAST attb	CW273	ATGCGGCCGCATGACAATATCGGGCGGCAATCA	Restriction digest via NotI + KpnI
		CW274	ATGGTACCTTATCCATTAAACACGCCCGTATCG	

Table S2: Transgenic flies generated in this study, Related to STAR Methods.

**Transgenic fly line**	**Vector used**	**Injected into**
UAS-circBtsz-exon	pUAST attb circBtsz-exon	y^1w^1; vas-phiC31; attP40
UAS-circBtsz-1000	pUAST-circBtsz-1000	y^1w^1; vas-phiC31; attP40
UAS-circBtsz-inv	pUAST-circBtsz-inv	y^1w^1; vas-phiC31; attP40
pWalium20-circBtsz-1	pWalium20-circBtsz-1	y^1w^1; vas-phiC31; attP40
UAS-circSfl-exon	pUAST attb circSfl-exon	y^1w^1; vas-phiC31; attP40
UAS-circSfl-1000	pUAST attb circSfl-1000	y^1w^1; vas-phiC31; attP40
UAS-circSfl-inv	pUAST attb circSfl-inv	y^1w^1; vas-phiC31; attP40
UAS-FLAG::circSfl-inv	pUAST attb FLAG::circSfl-inv	y^1w^1; vas-phiC31; attP40
pCFD4 Sfl N term	pCFD4 Sfl N term	y^1v^1; nanos-phiC31; attP40
FLAG::Sfl	pBS FLAG::Sfl	Progeny of nos-Cas9 x pCFD4 Sfl N term
pWalium20-circSfl-1	pWalium20-circSfl-1	y^1w^1; vas-phiC31; attP40
pWalium20-circSfl-2	pWalium20-circSfl-2	y^1w^1; vas-phiC31; attP40
pWalium20-circSfl-3	pWalium20-circSfl-3	y^1w^1; vas-phiC31; attP40

pCFD4 sfl$^{\Delta ex2}$	pCFD4 sfl$^{\Delta ex2}$	y^{1}v^{1}; nanos-phiC31; attP40
sfl$^{\Delta ex2}$	-	Progeny of nos-Cas9 x pCFD4 sfl$^{\Delta ex2}$
UAS-circSfl-ORF	pUAST attb circSfl-ORF	y^{1}w^{1}; vas-phiC31; attP40

Table S3: Oligonucleotides used for q-RT-PCR in this study, Related to STAR Methods.

**Target gene**	**Forward primer**	**Sequence (for)**	**Reverse primer**	**Sequence (rev)**
Rpl32	SOL268	ATATGCTAAGCTGTCGCACAAATGG	SOL269	GATCCGTAACCGATGTTGGGCA
circSfl	CW061	ATGTCGATACGGGCGTGTTT	CW062	CCAGACTGTCCACTCGCAAT
Sfl RA	CW170	ATTGAAGCTGTCGATTTGAGT	CW062	CCAGACTGTCCACTCGCAAT
Sfl RB	CW171	TGTGCGACAGATAAGAGGACG	CW062	CCAGACTGTCCACTCGCAAT
Sfl all	CW078	TCCCACGGCGATGTTATAGC	CW079	CAGATCCTTCAGTGCCCTCG
circ-Btsz	CW049	AGTCGGTGAGGCAGAGAGAT	CW050	CTGCTTTGCGGCCGAAATG
Btsz	CW049	AGTCGGTGAGGCAGAGAGAT	CW071	GGTTGTTGTGTGGCATCGAG
